# Engineered Salivary Peptides Reduce Enamel Demineralization Provoked by Cariogenic *S. mutans* Biofilm

**DOI:** 10.3390/microorganisms10040742

**Published:** 2022-03-30

**Authors:** Lina Maria Marin, Yizhi Xiao, Jaime Aparecido Cury, Walter Luiz Siqueira

**Affiliations:** 1 College of Dentistry, University of Saskatchewan, Saskatoon, SK S7N 5E4, Canada; lina.marin@usask.ca; 2 Schulich School of Dentistry, The University of Western Ontario, London, ON N6A 5C1, Canada; yxiao32@uwo.ca; 3 Piracicaba Dental School, University of Campinas, Piracicaba 13414-903, SP, Brazil; jcury@unicamp.br

**Keywords:** *Streptococcus mutans*, biofilm, dental caries, demineralization, acquired enamel pellicle, statherin, histatin, proteomics

## Abstract

Engineering of the acquired enamel pellicle using salivary peptides has been shown to be a promising anticaries strategy. However, the mechanisms by which these peptides protect teeth against tooth decay are not fully understood. In this study, we evaluated the effect of the engineered salivary peptides DR9-DR9 and DR9-RR14 on enamel demineralization in two experimental conditions: (1) adsorbed onto the enamel surface forming the AEP, and (2) forming the AEP combined with their use to treat the biofilms 2×/day, using a validated cariogenic *Streptococcus mutans* in vitro biofilm model. Biofilms were grown for 144 h on enamel slabs and then collected to determine the bacterial viability (CFU/biofilm) and biofilm mass (mg protein/biofilm), and to extract cellular/extracellular proteins, which were characterized by mass spectrometry. The culture medium was changed 2×/day to fresh medium, and pH (indicator of biofilm acidogenicity) and calcium concentration (indicator of demineralization) was determined in used medium. DR9-RR14 peptide significantly reduced enamel demineralization (*p* < 0.0001) in both experimental conditions. However, this peptide did not have a significant effect on biofilm biomass (*p* > 0.05) nor did it modulate the expression of cellular and extracellular bacterial proteins involved in biofilm cariogenicity. These findings suggest that DR9-RR14 may control caries development mainly by a physicochemical mechanism.

## 1. Introduction

Dental caries is a biofilm- and diet-dependent disease [1] that provokes a gradual dissolution of the dental mineral structure by the acids produced from dietary sugars by bacterial fermentation. The adsorption of selective salivary proteins and peptides to the dental surfaces precedes dental biofilm formation [2,3,4], forming an acellular film known as acquired enamel pellicle (AEP) [5]. The in vivo AEP is composed of around 130 different proteins [2], and approximately 50% are natural peptides [2,3,4]. These peptides are produced in the oral cavity after the proteolysis of salivary proteins by the action of salivary proteases of bacterial and human origin [6]. Amongst the natural peptides identified in the AEP, those originating from acidic proline-rich proteins (PRPs), histatin, and statherin, considered as the major pellicle precursor proteins [7], are the most abundant natural peptides composing the AEP [4].

The composition of the AEP determines its functions, and this knowledge may be useful to design strategies to control dental caries. On the one hand, proteinaceous components of the AEP have the ability to keep the homeostasis of the mineral composition of teeth by forming a semipermeable barrier on the tooth surface, reducing the demineralization of teeth provoked by acids produced in the biofilm after bacterial metabolism of carbohydrates [8,9,10,11,12,13]. Recently, it was demonstrated in in vitro studies using static caries models that the naturally occurring 9-residue phosphopeptide derived from statherin, known as DR9, limits the diffusion of acids throughout the AEP, reducing enamel demineralization [14]. On the other hand, modification of the proteinaceous components of the AEP may control the adhesion of certain pathogenic microorganisms to the teeth. In this regard, we recently demonstrated that histatin 3, and its functional 14-residue peptide known as RR14, display antimicrobial activity against planktonic *S. mutans* [14], the most cariogenic microorganism in the dental biofilm [15].

Salivary proteins respond to evolutionary pressure by the inclusion of functional domains into their primary structure, potentiating its activity [16]. Although neither statherin nor histatins contain duplicated or multiplicated sequences of amino acids, this knowledge was used to synthetically duplicate DR9, obtaining DR9–DR9, which has an enhanced ability to promote mineral homeostasis [14,17,18] and to inhibit *S. mutans* adherence to hydroxyapatite [19]. Another evolutionary process leads to the formation of functional complexes by certain salivary proteins once secreted into the oral cavity, protecting them against proteolysis, modulating their biological functions, and allowing the distribution of the proteins throughout the oral cavity [6]. The natural existence of proteinaceous complexes displaying multiple functions served as the basis for the development of the engineered hybrid peptide DR9-RR14 [14,17,18], which also displays antibacterial effect against planktonic *S. mutans* [14].

The knowledge of the functions exerted by histatin and statherin, and their natural and evolution-inspired engineered peptides, brings the possibility of using DR9–DR9 and DR9–RR14 peptides to control caries by interfering simultaneously with the dental biofilm formation and the physicochemical process of dental caries development, as previously suggested [14]. However, the caries-protective effect of these novel potential therapeutic agents and their mechanisms have not been investigated using in vitro caries models that consider the two main factors involved in the carious lesion development: biofilm accumulation on the dental surface and the frequent exposure to sucrose, the most cariogenic dietary fermentable sugar [20]. Thus, this study aimed at exploring the mechanisms by which the AEP engineered peptides DR9–DR9 and DR–RR14 control dental caries by using a validated *S. mutans* cariogenic in vitro biofilm model [21].

## 2. Materials and Methods

### 2.1. Experimental Design

A validated in vitro *S. mutans* cariogenic biofilm model [21] was used to test the effect of DR9–DR9 and DR9–RR14 on enamel demineralization in two experimental conditions: (1) adsorbed onto the enamel surface forming the AEP, and (2) forming the AEP combined with their use to treat the biofilms 2×/day. For each experimental condition, bovine enamel slabs (*n* = 12/group) were randomly allocated to one of the following treatments: statherin, histatin 3, DR9, DR9–DR9, DR9–RR14, RR14, 12,300 µg of fluoride (F^–^) per mL (positive control), or 50 mM NaCl pH 6.8 (negative control). The response variables were (1) pH of the culture medium, determined 2×/day as an indicator of the acidogenicity of developing biofilms; (2) cumulative calcium released from the enamel slabs, calculated from the concentration found in the culture medium, as a chemical indicator of enamel demineralization [22], and expressed in µg Ca/cm^2^ of enamel; and (3) biofilm biomass (CFU/biofilm and mg protein/biofilm), evaluated in eight of the biofilms collected on the sixth day of the experiment. In addition, cellular and extracellular proteome profiles were obtained after the mass spectrometry identification of the proteins extracted from four biofilms/group. Two independent assays per experimental condition were performed. Our hypothesis was that the engineered salivary peptides reduce enamel demineralization under highly cariogenic conditions, and it was tested at a significant level α of 5% (ANOVA and Tukey test).

### 2.2. Proteins and Peptides Tested

Synthetic statherin was purchased from Peptide Protein Research Ltd. (Hampshire, UK), while synthetic histatin 3 and peptides derived from statherin or histatin 3 were purchased from Synpeptide (Shanghai, China). All the proteins and peptides used in this study were previously described by Marin et al. [21]. Protein and peptide solutions were prepared in 50 mM NaCl pH 6.8 at a final concentration of 198 µM, 24 h before starting the experiment. The final concentration was checked in a UV-light spectrophotometer at a wavelength of 215 nm.

### 2.3. Enamel Slabs Preparation

Bovine enamel slabs were prepared and assembled as previously reported [21], and they were randomly distributed to each of the treatment groups.

### 2.4. Acquired Enamel Pellicle Formation

Enamel slabs were positioned in a 96-well culture plate and incubated for 24 h at 37 °C under constant agitation at 60 rpm/min with one of the following treatments (200 µL/well/slab): statherin, histatin 3, DR9, DR9–DR9, DR9–RR14, RR14, 12,300 µg F/mL, or 50 mM NaCl pH 6.8.

### 2.5. S. mutans Inoculation

After the AEP formation, enamel slabs were washed three times with 50 mM NaCl pH 6.8 and then transferred to a 24-well culture plate having the standard cell suspension (*S. mutans* UA159, 1 × 10^8^ CFU/mL) in 2 mL of tryptone yeast-extract broth (TYEB) buffered 10× (to avoid pH dropping and enamel demineralization during the adhesion period) and supplemented with 1% glucose. The slabs were incubated for 8 h at 37 °C and 10% CO_2_ to allow bacterial adhesion, then washed three times with 0.9% NaCl, transferred to fresh TYEB supplemented with 0.1 mM glucose, and incubated for 16 h at 37 °C and 10% CO_2_.

### 2.6. Cariogenic Biofilm Development

#### 2.6.1. Experimental Condition 1

**Figure 1:**
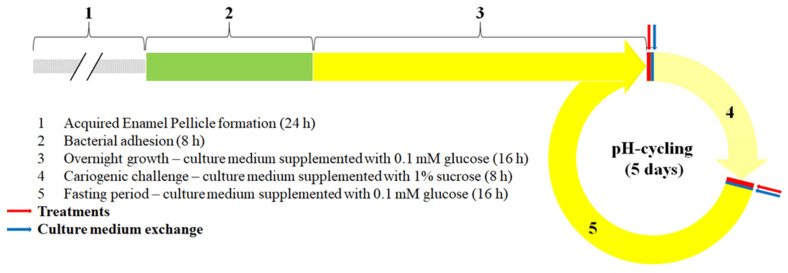
Outline of the cariogenic *S. mutans* biofilm experiments done for experimental conditions 1 and 2. (1) Enamel slabs were incubated with treatment solutions for 24 h to form the AEP. (2) Peptide-coated enamel slabs were inoculated with *S. mutans* in culture medium supplemented with 1% glucose, and the bacteria were allowed to adhere for 8 h. (3) Enamel slabs rested overnight in culture medium supplemented with 0.1 mM glucose. Cariogenic biofilms were formed by repeating the daily pH-cycling regimen depicted in the diagram (steps 4 and 5), with the culture medium being replaced twice per day for fresh medium (blue lines and arrows): (4) Cariogenic challenge was done by feeding the bacteria with 1% sucrose for 8 h/day. (5) Bacteria fasted overnight in culture medium having 0.1 mM glucose. Daily treatments (red lines and arrows) were done 2×/day before and after the cariogenic challenges only for experimental condition 2.

After inoculation of *S. mutans* and at the 24 h time point, slabs were exposed to 1% sucrose in TYEB for 8 h at 37 °C and 10% CO_2_, as a daily constant cariogenic challenge. After 8 h, the culture medium was replaced by fresh TYEB supplemented with 0.1 mM glucose and biofilms rested overnight at 37 °C and 10% CO_2_. These procedures were repeated during the following days until the biofilms completed 144 h of formation (Figure 1).

#### 2.6.2. Experimental Condition 2

The procedure to develop cariogenic biofilms described above was repeated, but in this experimental condition biofilms were additionally treated daily with the corresponding proteins/peptides solutions. Treatments were done with the same solutions used to form the AEP (except for the positive control group, which was treated with a solution containing 275 µg F^–^/mL, simulating the dilution that occurs in the saliva while tooth brushing with a toothpaste containing 1100 µg F^–^/g [23]). Treatment solutions were prepared fresh daily. Treatments were performed 2×/day, in the morning, after 16 h of incubation in TYEB supplemented with 0.1 mM glucose, and after 8 h of the cariogenic challenge with 1% sucrose. For this, the biofilms were removed from the culture medium, washed three times with 0.9% NaCl, transferred to a 96-well plate containing 200 µL of the corresponding treatment solution, and incubated for 10 min at room temperature. After the treatment period, the slabs were washed three times with 0.9% NaCl and finally transferred to the corresponding fresh culture medium (Figure 1).

### 2.7. Culture Medium Analyses

The replaced culture medium was used to assess its pH, as an indicator of acidogenesis by the developing biofilms, and to determine the calcium concentration in the culture medium, as a chemical indicator of enamel demineralization [22]. The acid production by the bacteria in the biofilm was analyzed 2×/day by determining the pH of the culture medium (immediately after each cariogenic challenge and in the following morning, after 16 h of incubation). For this, a pH electrode connected to a pH meter with a pH measurement resolution of ±0.01, previously calibrated with pH 4.0 and 7.0 standards, was used. After pH determinations, an aliquot from each well was transferred to a microcentrifuge tube and stored at −80 °C for calcium analysis.

For calcium analysis, 25 μL of the culture medium from each well was used to quantify the calcium concentration (mM) of it, using the Arzenazo III colorimetric method [24], and solutions with known concentrations of calcium as standards (0–1.2 mM). The variation coefficient of the repeated analyses (duplicate) was 1.9%. In addition, aliquots of the fresh culture medium were stored every day to determine the basal calcium concentration of them. Absorbance readings were performed at 650 nm on a 96-well plate spectrophotometer reader. The cumulative concentration of calcium in the culture medium (µg Ca/cm^2^) was determined by the sum of the concentrations found in the last three days of exposure to sucrose (time points: 80 h, 104 h, and 128 h), representing the sum of the total quantity of calcium released from enamel to the medium until biofilms were harvested. This analysis of enamel demineralization was done because previous studies showed that there is a correlation between the analysis of enamel demineralization done by microhardness test (surface hardness loss) and the chemical analysis of demineralization (cumulative calcium released from the enamel), validating its use [25,26].

### 2.8. Biofilm Harvesting and Analyses

In the morning of the last day of biofilm formation, when the experiment completed 144 h, biofilms were washed three times with 0.9% NaCl. Biofilm biomass and cell viability were determined in eight enamel slabs randomly selected from each group. The remaining four biofilms were used to extract the extracellular matrix (ECM) and cellular proteins.

For biofilm mass and bacterial viability analyses, the corresponding biofilms were individually transferred to microcentrifuge tubes containing 1 mL of 0.9% NaCl and sonicated at an amplitude of 10% (sonic dismembrator, model 500, Fisher Scientific, Pittsburgh, USA) for 30 s on ice to separate the bacteria from the enamel slabs. An aliquot of 200 μL of the cell suspension was transferred to a microcentrifuge tube to which 200 μL of 2 M NaOH was added. Alkali-soluble protein concentration (mg protein/biofilm) was determined (BCA, Thermo Scientific) in the supernatant obtained after incubating the suspension for 15 min at 100 °C and centrifuging it at 10,000× *g* for 10 min at 4 °C. The determination of biofilm biomass by the analysis of total alkali soluble protein concentration was selected because there is a correlation between these two variables, validating its use [27]. For the assessment of bacterial viability, we standardized the method of resazurin reduction using 50 μL of the cell suspension that were transferred in duplicate to a well of a 96-well plate, to which 50 μL of KCl buffer (0.05 M KCl, 1 mM CaCl_2_, and 0.1 mM MgCl_2_) and 100 μL of a solution containing 2% resazurin in KCl buffer and 0.5% glucose, was added. Fluorescence intensity was measured with a fluorescence spectrophotometer (excitation wavelength: 560 nm, emission wavelength: 590 nm, model 650-40, Hitachi, Tokyo, Japan) after a 90-min incubation period at 37 °C. Bacterial viability was determined using a calibration curve of fluorescence intensity versus cell viability (CFU/biofilm) performed in triplicate on the same day from serial dilutions of one of the biofilms.

For extracellular matrix (ECM) and cellular proteins extraction, the corresponding biofilms were collected and transferred to microcentrifuge tubes containing 500 μL of 0.1 N NaOH and 1 mM EDTA (for each 10 mg of biofilm wet weight) [28,29]. Biofilms were incubated for 1 h at 4 °C under constant agitation and then centrifuged at 10,000× *g* for 10 min at 4 °C. The supernatant having the ECM proteins was transferred to a microcentrifuge tube, and the proteins were precipitated by adding three volumes of ice-cold acetone [28,29]. The pellet containing the bacteria was processed to extract the cellular proteins by adding 500 μL of 0.1 N NaOH and 1 mM EDTA. The tube was vortexed, incubated for 15 min at 100 °C, and centrifuged at 10,000× *g* for 10 min at 4 °C. The supernatant containing the cellular proteins was transferred to a microcentrifuge tube, and the proteins were precipitated and concentrated with three volumes of ice-cold acetone. The total protein concentration was determined in duplicate by the bicinchoninic acid assay using a spectrophotometer at a wavelength of 562 nm. An amount of protein equivalent to 10 μg was digested with trypsin (5% trypsin (*v/v*) in 50 mM NH_4_HCO_3_, pH 7.8) for 16 h at 37 °C and purified with a C18 column for proteomic analysis. Subsequently, the samples were diluted in a buffer containing 80% acetonitrile/19.9% H_2_O/0.1% trifluoroacetic acid (*v/v*), dried, and reconstituted in 10 µL of 97.5% H_2_O/2.4% acetonitrile/0.1% formic acid (*v/v*) and were subsequently subjected to reverse-phase LC–ESI-MS/MS for proteomics analysis.

### 2.9. Proteomics Analyses

The analysis of tandem mass spectrometry of the ECM and cellular proteins was performed according to a protocol previously described [19] using a Velos LTQ (Thermo-Finnigan, San Jose, CA, USA). The obtained MS/MS spectra were searched against *S. mutans* protein databases (Swiss Prot and TrEMBL, Swiss Institute of Bioinformatics, Geneva, Switzerland, http://ca.expasy.org/sprot/, accessed on 2 April 2019) using the SEQUEST algorithm in Proteome Discoverer software, version 1.3 (Thermo Scientific, San Jose, CA, USA). Search results were filtered for a False Discovery Rate of 1% employing a decoy search strategy utilizing a reverse database. An additional inclusion criterion for the positive identification of proteins was that the same protein passed the filter score in at least three different MS analyses from the same group in a total of three mass spectrometry analyses per group. The following biological processes were used to classify the identified proteins: adaptive responses to environmental changes; bacterial adherence and biofilm formation; carbohydrate metabolism and energy production; cell division, replication, and cell wall synthesis; nucleoside/nucleotide metabolism and synthesis; transcription; translation and protein synthesis; transport; other metabolic processes; and uncharacterized/unknown proteins. The relative percentage of the proteins identified per biological function was calculated from the total proteins found in each treatment group. This variable was used to estimate those biological functions in which the proteins were down- or upregulated when the relative percentage was twice higher or lower than the negative control, respectively.

### 2.10. Statistical Analyses

The normal distribution of the errors and the homogeneity of the variances were checked for all response variables tested. Those variables that did not satisfy the assumptions were transformed. Once these assumptions were fulfilled, analyses, of variance (ANOVA) were performed to test the effect of the treatments on the response variables. Tukey tests were performed when an effect was observed for each response variable. All statistical analyses were done at a significant level α of 5%, using SPSS (IBM SPSS Statistics for Windows, Version 21.0. Armonk, NY, USA: IBM Corp.).

## 3. Results

### 3.1. Acidogenesis by S. mutans Biofilms

**Figure 2:**
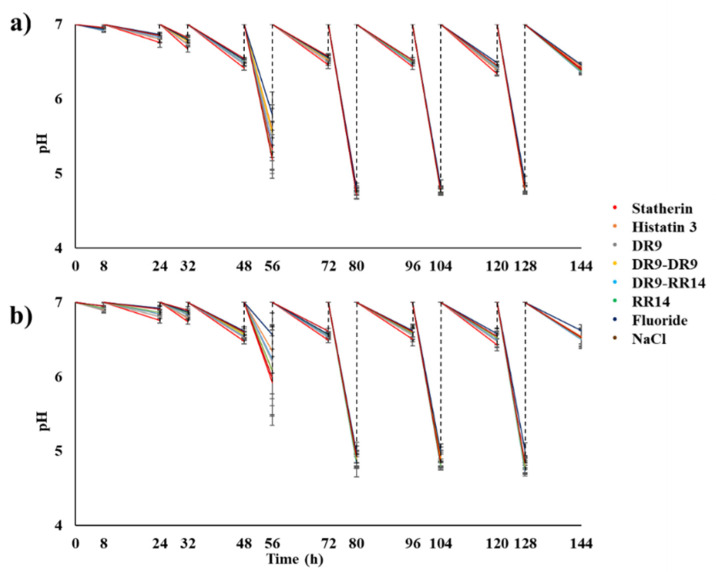
pH of the culture medium according to the treatments as a function of time. (**a**) pH of the culture medium obtained when the peptides were present only in the AEP (experimental condition 1). (**b**) pH of the culture medium found when the peptides were used both to form the AEP and to treat the biofilms (experimental condition 2). Mean ± S.D., *n* = 12.

The results of the study in which the effect on enamel demineralization of the engineered salivary peptides, present only in AEP, was evaluated, showed a decrease in the pH of the culture medium after the cariogenic challenges, these drops being detectable from the third day of the experiment (56 h). At this time point it was possible to observe statistical differences among the treatment groups (ANOVA and Tukey test, *p* < 0.0001), with the lowest pH values (below 5.5) observed in the groups treated with NaCl, RR14, statherin, DR9–RR14, and DR9; whereas the highest values were obtained in the groups treated with fluoride, histatin 3, and DR9–DR9 (above 5.5). At 56 h, the group treated with fluoride was the one with the lowest acidogenicity, and the group treated with NaCl, as the negative control, was the one with the highest production of acids. From the fourth day of the experiment, pH values were similar among groups, with the pH being lower than 5.0 (Figure 2a). Comparable results were obtained when the biofilms were additionally treated on a daily basis, but no statistical differences were observed at any time among groups (ANOVA and Tukey test, *p* > 0.05) (Figure 2b).

### 3.2. Enamel Demineralization

**Figure 3:**
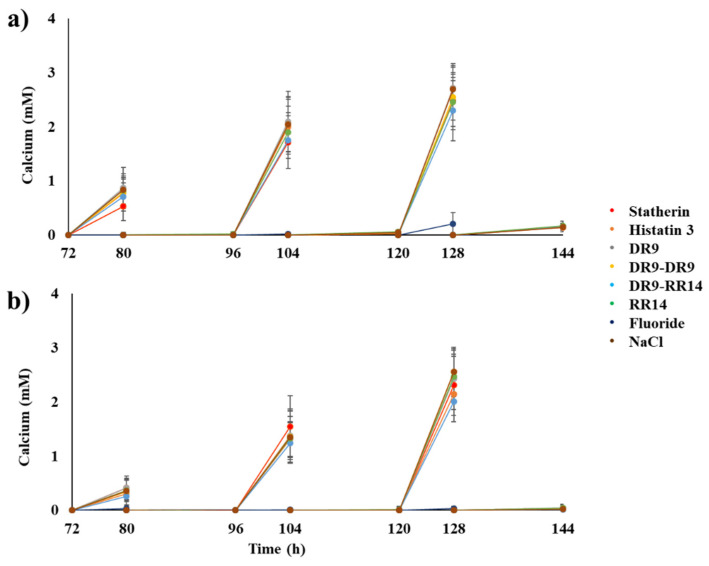
Calcium concentration in the culture medium according to the treatments. Concentration found after the cariogenic challenge carried out on the fourth (80 h), fifth (104 h), and sixth (128 h) days, and in the last three days after the overnight fasting period (72 h, 96 h, 120 h, and 144 h). (**a**) Calcium concentration in the culture medium obtained when the peptides were present only in the AEP (experimental condition 1). (**b**) Calcium concentration in the culture medium found when the peptides were used both to form the AEP and to treat the biofilms (experimental condition 2). Mean ± S.D., *n* = 12.

The profile of the calcium concentration in the culture medium as a function of the time of biofilm formation, showed, for all treatment groups, an inverse relation with the pH, since the lower the pH, the higher the calcium concentration, indicating a demineralization of the enamel due to the production of acids by the bacteria in the biofilm (Figure 3). However, the calcium released from the enamel was only detectable after the third day of the experiment (80 h) (Figure 3), this being the time point where the pH of the culture medium was lower than 5.0 in all the treatment groups (Figure 2). In the profile of the calcium concentration as a function of time, it was possible to observe that enamel demineralization was almost completely inhibited by fluoride, at all time points evaluated (Figure 3). On the one hand, when the salivary proteins/peptides were used only to form the AEP, there was a reduction in the demineralization only in the groups treated with statherin and DR9–RR14 at 80 h, with these values being statistically different from the other groups (ANOVA and Tukey test, *p* < 0.05). Although the group treated with the peptide DR9–RR14 showed a lower demineralization at the 104 h and 128 h time points, there were no statistically significant differences among the groups treated with salivary proteins/peptides (*p* > 0.05) (Figure 3a). On the other hand, when the biofilms were additionally treated with the salivary proteins/peptides daily, there were no differences among groups at 80 h and 104 h. At 128 h, there was a significant reduction of enamel demineralization in the slabs treated with DR9–RR14, with the calcium concentration being equivalent to that obtained in the groups treated with histatin 3 and statherin (ANOVA and Tukey test, *p* < 0.05) (Figure 3b).

**Figure 4:**
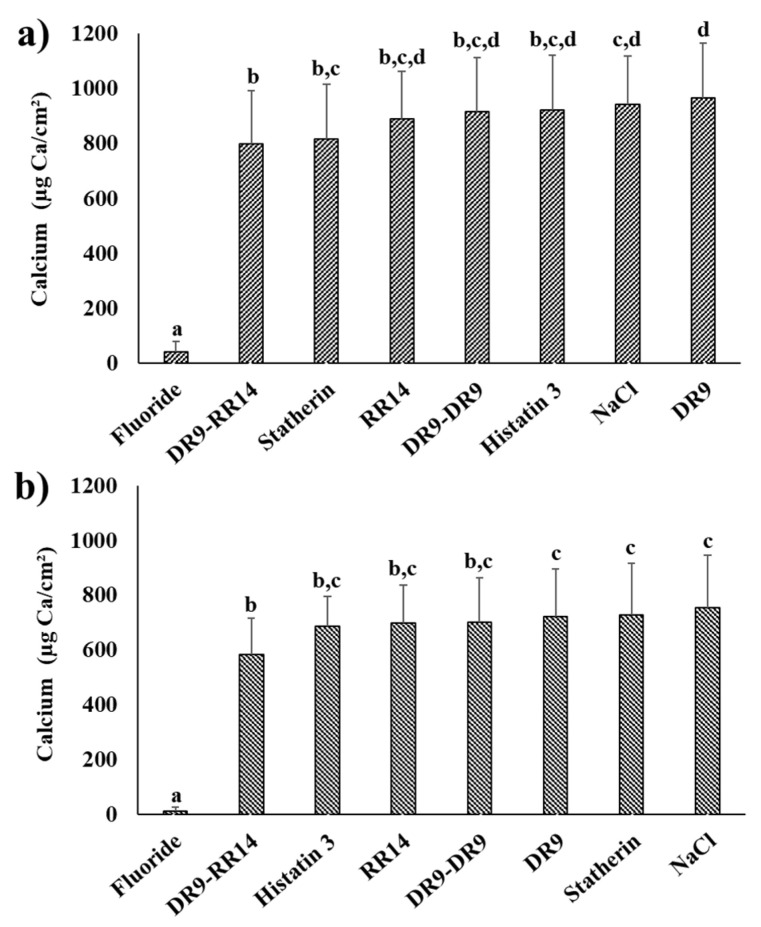
Cumulative calcium released from enamel into the culture medium (µg Ca/cm^2^) according to the treatments. (**a**) Cumulative calcium concentration in the culture medium obtained when the peptides were present only in the AEP (experimental condition 1). (**b**) Cumulative calcium concentration in the culture medium found when the peptides were used both to form the AEP and to treat the biofilms daily (experimental condition 2). For statistical analysis, cumulative calcium concentration was transformed to its square root. Means followed by distinct lowercase letters show statistical differences among groups (ANOVA and Tukey test, *p* < 0.0001; Mean ± S.D., *n* = 12).

Regarding the cumulative calcium concentration (µg Ca/cm^2^) in the culture medium, significant differences were found among all treatment groups, with a lower demineralization found in the enamel slabs treated with fluoride, followed by the group treated with the hybrid peptide DR9–RR14 (Figure 4). When the salivary proteins/peptides were used only to form the AEP, the demineralization obtained in the group treated with DR9–RR14 was similar to that of statherin, RR14, DR9–DR9, and Histatin 3; while the groups treated with NaCl and DR9 were those that had the highest demineralization values, being different from the two groups that demonstrated the highest protection against enamel demineralization (fluoride and DR9–RR14) (ANOVA and Tukey test, *p* < 0.0001) (Figure 4a). After using the salivary proteins/peptides to also treat the biofilms daily, the calcium concentration found in the group treated with DR9–RR14 was similar to that of the groups treated with histatin 3, DR9–DR9, and RR14. Those groups treated with DR9, statherin, and NaCl were among those that showed the highest demineralization, being different from those that showed the highest protection against demineralization (fluoride and DR9–RR14) (ANOVA and Tukey test, *p* < 0.0001) (Figure 4b).

### 3.3. S. mutans Biofilm Biomass and Bacterial Viability

Concerning the biomass analyses, the data showed a significant reduction of biofilm mass and bacterial viability in the positive control group treated with fluoride when compared to the other groups (Table 1 and Table 2, columns 3 and 4). In addition, the groups treated with NaCl, RR14, and DR9–RR14 displayed the highest biofilm biomass when the peptides were used only to form the AEP (Table 1, column 3).

### 3.4. S. mutans Cellular and Extracellular Proteome Profiles

**Figure 5:**
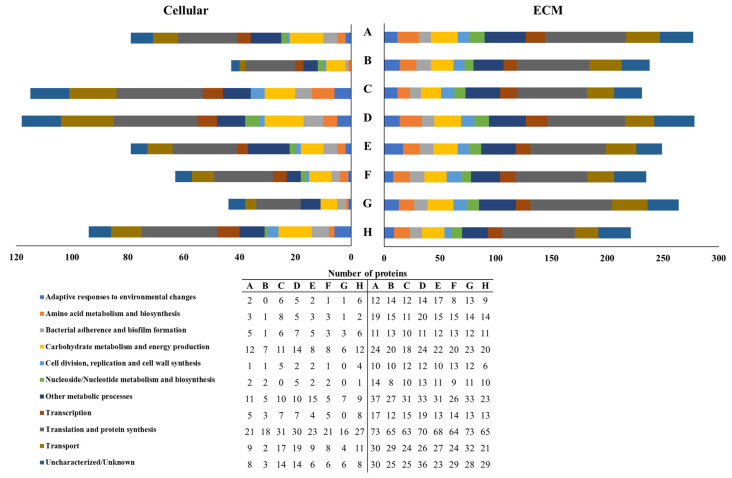
Distribution of *S. mutans* cellular and ECM proteins into specific biological processes according to the treatments done to form the AEP in the experimental condition 1. Statherin (**A**), histatin 3 (**B**), DR9 (**C**), DR9–DR9 (**D**), DR9–RR14 (**E**), RR14 (**F**), fluoride (**G**), NaCl (**H**).

**Figure 6:**
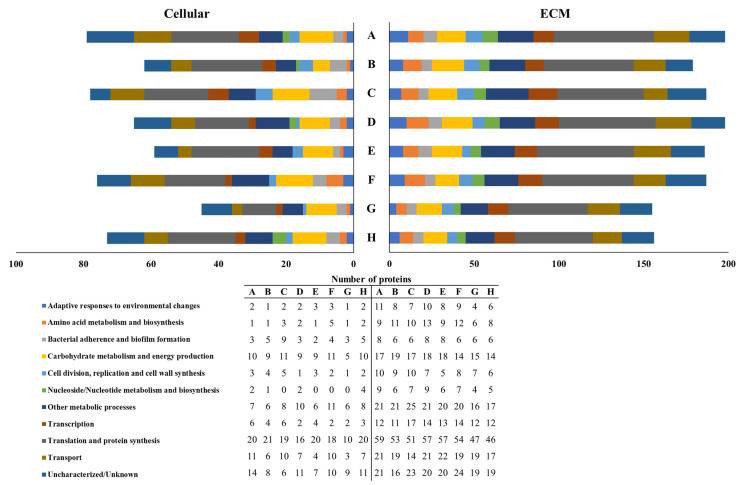
Distribution of *S. mutans* cellular and ECM proteins into specific biological processes according to the treatments done to form the AEP and used daily in the experimental condition 2. Statherin (**A**), histatin 3 (**B**), DR9 (**C**), DR9–DR9 (**D**), DR9–RR14 (**E**), RR14 (**F**), fluoride (**G**), NaCl (**H**).

The proteome profiles obtained after generally classifying the identified cellular and ECM proteins by the biological process in which each protein participates showed that the proteins/peptides used both to form the AEP (Figure 5) and to treat the biofilms daily (Figure 6) modulate the expression of cellular proteins, while the proteome profiles obtained from the ECM proteins were similar among groups. When the treatments were done to only form the AEP, the group treated with histatin 3 did not express proteins participating in adaptive responses to environmental changes, while in the group treated with DR9, any protein participating in nucleoside/nucleotide metabolism and synthesis was detected. Additionally, fluoride treatment nullified the expression of proteins participating in the following biological functions: cell division, replication, and cell wall synthesis; nucleoside/nucleotide metabolism and synthesis; and transcription (Figure 5). Moreover, the addition of the daily treatments only abolished the expression of proteins involved in nucleoside/nucleotide metabolism in the groups treated with DR9, DR9–RR14, RR14, and fluoride (Figure 6).

Despite the differences previously described, in both experiments, the relative percentage of the cellular and ECM proteins classified per biological process were similar among groups (Figure 5 and Figure 6). On the one hand, the most abundant cellular proteins were those proteins involved in translation and protein synthesis (28.9 ± 5.2%); carbohydrate metabolism and energy production (13.2 ± 1.8%); uncharacterized/unknown proteins (12.3 ± 4.1%); other metabolic processes (11.8 ± 3.3%); and transport (10.9 ± 3.1%). The less abundant proteins were those participating in nucleoside/nucleotide metabolism and synthesis (1.9 ± 1.9%); adaptive responses to environmental changes (3.0 ± 1.6%); cell division, replication, and cell wall synthesis (3.0 ± 1.8%); amino acid metabolism and biosynthesis (3.3 ± 1.7%); transcription (5.6 ± 2.3%); and bacterial adherence and biofilm formation (5.9 ± 2.1%). On the other hand, the most abundant ECM proteins were those proteins participating in translation and protein synthesis (28.3 ± 1.5%); other metabolic processes (11.6 ± 1.1%); uncharacterized/unknown proteins (11.3 ± 1.3%); transport (10.6 ± 1.2%); and carbohydrate metabolism and energy production (8.9 ± 0.7%) (Table 3). The less abundant proteins were those involved in nucleoside/nucleotide metabolism and synthesis (4.0 ± 0.7%); bacterial adherence and biofilm formation (4.2 ± 0.7%); cell division, replication and cell wall synthesis (4.3 ± 0.9%); adaptive responses to environmental changes (4.6 ± 1.0%); amino acid metabolism and biosynthesis (5.8 ± 0.9%); and transcription (6.5 ± 1.1%) (Table 3).

The expression of cellular proteins participating in the following biological functions was found to be either down- or upregulated at least twice with respect to the negative control, especially when the treatments were done only to form the AEP: amino acid metabolism and biosynthesis; cell division, replication, and cell wall synthesis; and nucleoside/nucleotide metabolism and synthesis (Table 3). Although the proteins that promote adaptive responses to environmental changes were downregulated, other bacterial proteins directly related to the cariogenicity of the biofilms like those participating in bacterial adherence and biofilm formation, and carbohydrate metabolism and energy production, were not modulated by the engineered peptides (Table 3).

The list of the proteins identified in each of the experiments is included as supplementary information (Supplementary Tables S1–S4).

## 4. Discussion

The results from this study showed that the hybrid peptide DR9–RR14 displayed a potential protective effect against enamel demineralization when adsorbed onto the enamel, forming the AEP, before the formation of cariogenic biofilms (Figure 4a). The anticaries effect observed in the group treated with the peptide DR9–RR14 was significantly different from the negative control but similar to that obtained when the enamel was treated with statherin, DR9–DR9, RR14, and histatin 3. However, the protection against enamel demineralization conferred by the latter groups was statistically similar to that obtained with the negative control (Figure 4a), indicating that only DR9–RR14 was able to reduce enamel demineralization when used to form the AEP before the development of cariogenic biofilms. However, calcium concentration found daily during biofilms development (Figure 3) suggests that statherin, at 80 h and 104 h, and DR9–RR14 at 104 h and 128 h, were able to reduce the demineralizing effect of the acids produced by the bacteria in the biofilm between 15% to 36%, respectively, in comparison to the negative control. The mechanism by which the DR9–RR14 peptide exerts the anticaries effect cannot be explained by the antibacterial effect observed in planktonic *S. mutans* [14], since both the biomass and the bacterial viability were similar to those values found in the other groups, except for the positive control (fluoride) (Table 1). However, the pH values obtained up to 56 h suggest a possible effect on the reduction of bacterial metabolism (Figure 2) since the bacteria treated with histatin 3, DR9, and DR9–DR9 produced fewer acids than the other groups. This possible effect was eliminated as soon as biofilm cariogenicity increased when the biofilms became mature, or by the eventual release of adsorbed peptides onto the enamel surface during acid production or biofilm manipulation.

DR9–RR14 peptide adsorbed onto the enamel may potentially be controlling the caries process by limiting the formation of enamel dissolution foci [30,31,32]. By doing so, this peptide reduces the diffusion of acids from the biofilm to the enamel mineral, or limits the transport of the dissolved dental mineral ions into the culture medium, as previously observed when the AEP was formed from total saliva [12]. Considering the structural analysis of the interaction between statherin and HAp [33], we can predict that the two phosphoserines in the 2nd and 3rd positions of the DR9–RR14 peptide are responsible for stabilizing the protein/mineral interaction, while the positively charged amino acids in the 6th, 9th, 10th, 11th, 15th, 20th, and 23rd positions interact with phosphate through electrostatic interactions [34]. Furthermore, the prediction of the secondary structure of DR9–RR14 peptide suggests an α-helix formation from the amino acids in the 3rd to the 15th positions [35], which could be limiting the enamel demineralization process occurring under this structure [33,36,37] and promoting mineral homeostasis [32]. The same mechanism may explain the mild caries-protective effect observed in the group treated with the duplicated peptide DR9–DR9, which contains four phosphorylated amino acids in positions 2, 3, 10, and 11 [14] and forms two α-helixes from the amino acids in the positions 4th to the 9th, and 13th to 16th [35], justifying the potential anticaries effect also previously demonstrated in a static model using demineralizing solutions [14]. The hypothesis that the caries-protective effect displayed by DR9–RR14 peptide is due to a physicochemical protection is supported by the fact that the expression of cellular and ECM proteins involved in critical processes related to the cariogenicity of the biofilms were not affected by the engineered peptides nor by any other protein/peptide used in this study (Table 3, Figure 5). These results also suggest that the inhibitory effect of DR9–DR9 peptide on *S. mutans* adherence [19] disappears when biofilms are grown in highly cariogenic environments, as is the case with the cariogenic biofilm model used in this study [21].

Contrary to what was expected, the anticaries effect of the engineered peptides did not increase when biofilms were additionally treated daily with them (experimental condition 2, Figure 4b). In this experimental design we simulated the daily use of a mouthwash for the delivery of the peptides to the oral cavity, expecting to replace the peptides that might be released from the dental surface to the culture medium over time. In these experiments, we obtained the same trend of reduction of demineralization observed when the peptides were used only to form the AEP (experimental condition 1); the peptide DR9–RR14 showing a significant potential anticaries effect (Figure 4b). Surprisingly, the group treated with statherin was one of the groups displaying higher mineral loss, similar to that obtained in the negative control (Figure 4b). These results suggest that the peptides in aqueous suspension (50 mM NaCl, pH 6.8) were not able to penetrate through the biofilms and may have been retained at the biofilm surface by binding to the ECM components and bacteria on the external surface of the biofilms [38]. Due to the high growth rate of the bacteria in the biofilm during the exposure to sucrose, the bacteria-bound molecules could have acted as bridges among bacterial cells, favoring the growth of biofilms, explaining why these biofilms were thicker than those formed in the absence of the daily treatments (Table 2, column 3). In case these peptides/proteins displayed an antimicrobial effect on the bacteria in the external surface of the biofilms, it could have been neutralized by the fast bacterial growth rate favored by sucrose metabolism, justifying the fact that both the biomass and cell viability did not differ among groups treated with these molecules (Table 2). These findings are also supported by the fact that most of the cellular and ECM proteins participating in each of the biological functions were similarly identified in all treatment groups (Supplementary Tables S3 and S4), confirming that the bacteria were metabolically active and efficiently growing in the biofilms until the last day of the experiment, when biofilms were harvested. As observed when the peptides were only in the AEP, there was a trend for the means of the pH values obtained until the third day of the experiment (72 h) to be different among groups (Figure 2b). Although these differences were not statistically significant, there were fewer acids being produced by the bacteria in the biofilms when they were treated with histatin 3 and its derived peptides RR14 and DR9–RR14, suggesting a possible antimicrobial effect exerted by these molecules only on young biofilms (Figure 2b). The encapsulation of the engineered peptides in nanoparticles may be a strategy to release them at the tooth/biofilm interface when the cariogenic biofilms mature and carious lesions are likely to be developed, which should be further investigated.

In all the experiments, fluoride almost completely inhibited enamel demineralization (Figure 4). This result was expected because a fluoridated solution containing 12,300 µg F/g was used, simulating the topical application of acidified fluoride gel to form a layer of crystals similar to calcium fluoride (CaF_2_-like) covering the enamel surface [39], in the same way that the salivary proteins cover the teeth by forming a proteinaceous film onto dental surfaces [40]. The inhibition of enamel demineralization caused by fluoride is due to the high amount of these CaF_2_-like crystals formed onto the enamel surface (188.2 ± 77.1 µg F/cm^2^ enamel, data not shown) during the 24 h application period used in this study [41,42]. CaF_2_-like reservoirs are solubilized slowly, continuously releasing fluoride to the biofilm fluid [43], making it available to interfere with the caries process [20] and explaining the inhibition of demineralization observed in our experiments (Figure 4). Furthermore, fluoride delivered from the dissolution of the CaF_2_-like crystals may have had an antimicrobial effect on *S. mutans*, since fluoride reduces biofilm biomass and bacterial viability [44], as observed in this study (Table 1 and Table 2). To support this observation, we also determined fluoride concentration in the culture medium over the time of the experiment and in the biofilm collected at 144 h (data not shown). The results showed that during the adherence phase (first 24 h), 40 µg F/g was found in the culture medium, indicating that this high concentration of fluoride had a bacteriostatic effect, inhibiting bacterial adherence to the enamel [45]. Then, biofilms started to form slowly during the following days of the experiment, where the fluoride in the culture medium was ~1 µg F/g, which is expected to not exert a bacteriostatic effect on *S. mutans* [46]. Fluoride may have also impaired biofilm formation and metabolism, demonstrated by the proteome analysis of the cellular compartment from bacteria treated with fluoride (Table 3).

Concerning the methodological aspects of this study, the use of a single-species biofilm model was selected because *S. mutans* is considered the most cariogenic microorganism found in the dental biofilm (15) due to its acidogenicity, aciduricity [47], and the ability to synthesize extracellular polysaccharides from sucrose metabolism [48,49,50], and the use of this bacterium is considered appropriate for the study of dental demineralization [22]. For the same reasons, sucrose was selected because it is considered as the most cariogenic carbohydrate from the diet [50]. Moreover, the cariogenic in vitro biofilm model used can be classified as a cycling model, which better mimics the in vivo caries process, appropriately simulating the demineralization and remineralization processes that occur at the tooth–biofilm interface when exposed to fermentable carbohydrates from the diet (Figure 1 and Figure 2) [21]. The slight but significant protection against demineralization conferred by DR9–RR14 peptides, when highly cariogenic conditions were simulated, suggests that its anticaries effect may increase if cariogenic biofilms are grown under low cariogenic conditions (i.e., exposure to sucrose 2 h or 4 h per day), which should be further investigated. Regarding the treatments, the carious protective effect of the combination of the engineered peptides in the AEP with the daily use of fluoride was not evaluated in this study, the result of which would be highly relevant considering the effectiveness of fluoride in the control of the caries process [51,52]. The use of single peptide solutions in the absence of saliva could be considered as one of the limitations of this study since the possible interaction between the salivary components and our engineered peptides during the formation of the AEP was not simulated [53], which could influence the observed caries-protective effect. However, the results of this study, using a single component approach of the AEP and a *S. mutans* cariogenic biofilm model, suggested that our evolution-inspired engineered salivary peptide DR9–RR14 exerts the anticaries effect by providing a physicochemical protection against enamel demineralization. The promotion of the mineral homeostasis of teeth provided by DR9–RR14 represents a new translational approach for the prevention/treatment of dental caries.

## Figures and Tables

**Table 1:** Biomass (mg protein/biofilm) and bacterial viability (CFU/biofilm) according to the treatments done to form the AEP in the experimental condition 1.

Treatments	mg Protein/Biofilm	CFU/Biofilm
	Mean (±S.D.)	Mean (±S.D.)
Fluoride	0.77 (0.07) ^a^	4.97^10^ (7.68^9^) ^a^
Histatin 3	0.96 (0.09) ^b^	6.94^10^ (9.62^9^) ^b^
Statherin	1.04 (0.14) ^b.c^	6.96^10^ (1.15^10^) ^b^
DR9–DR9	1.04 (0.10) ^b.c.d^	6.99^10^ (9.95^9^) ^b^
DR9	1.06 (0.11) ^b.c.d^	7.02^10^ (6.41^9^) ^b^
NaCl	1.10 (0.11) ^c.d^	7.10^10^ (9.89^9^) ^b^
RR14	1.11 (0.11) ^c.d^	7.13^10^ (5.33^9^) ^b^
DR9–RR14	1.16 (0.09) ^d^	7.42^10^ (5.77^9^) ^b^

Means followed by distinct lowercase letters show statistical differences among groups (ANOVA and Tukey test, *p* < 0.001).

**Table 2:** Biomass (mg protein/biofilm) and bacterial viability (CFU/biofilm) according to the treatments done to form the AEP and used daily in the experimental condition 2.

Treatment	Mg Protein/Biofilm	CFU/Biofilm
	Mean (±S.D.)	Mean (±S.D.)
Fluoride	1.08 (0.18) ^a^	3.67^10^ (1.42^10^) ^a^
NaCl	1.38 (0.19) ^b^	7.45^10^ (1.52^10^) ^b^
Histatin 3	1.40 (0.17) ^b^	7.57^10^ (1.19^10^) ^b^
Statherin	1.42 (0.16) ^b^	7.44^10^ (1.10^10^) ^b^
DR9	1.42 (0.19) ^b^	7.23^10^ (1.44^10^) ^b^
DR9–DR9	1.43 (0.17) ^b^	7.64^10^ (1.10^10^) ^b^
DR9–RR14	1.45 (0.20) ^b^	7.72^10^ (1.39^10^) ^b^
RR14	1.50 (0.18) ^b^	7.34^10^ (1.39^10^) ^b^

Means followed by distinct lowercase letters show statistical differences among groups (ANOVA and Tukey test, *p* < 0.001).

**Table 3:** Relative percentage of *S. mutans* cellular proteins classified by the biological function according to the experimental design.

Experimental Design	AEP only (Experimental Condition 1)								AEP and Daily Treatment (Experimental Condition 2)								Mean	S.D.
Biological Function	Treatment								Treatment									
	A	B	C	D	E	F	G	H	A	B	C	D	E	F	G	H		
Adaptive responses to environmental changes	2.5	0.0	5.2	4.2	2.5	1.6	2.3	6.4	2.5	1.5	2.5	3.1	5.1	3.9	2.4	2.7	3.0	1.6
Amino acid metabolism and biosynthesis	3.8	2.3	7.0	4.2	3.8	4.8	2.3	2.1	1.3	1.5	3.8	3.1	1.7	6.6	2.4	2.7	3.3	1.7
Bacterial adherence and biofilm formation	6.3	2.3	5.2	5.9	6.3	4.8	6.8	6.4	3.8	7.6	11.4	4.6	3.4	5.3	7.3	6.8	5.9	2.1
Carbohydrate metabolism and energy production	15.2	16.3	9.6	11.9	10.1	12.7	13.6	12.8	12.7	13.6	13.9	13.8	15.3	14.5	12.2	13.5	13.2	1.8
Cell division, replication, and cell wall synthesis	1.3	2.3	4.3	1.7	2.5	1.6	0.0	4.3	3.8	6.1	6.3	1.5	5.1	2.6	2.4	2.7	3.0	1.8
Nucleoside/Nucleotide metabolism and biosynthesis	2.5	4.7	0.0	4.2	2.5	3.2	0.0	1.1	2.5	1.5	0.0	3.1	0.0	0.0	0.0	5.4	1.9	1.9
Other metabolic processes	13.9	11.6	8.7	8.5	19.0	7.9	15.9	9.6	8.9	9.1	10.1	15.4	10.2	14.5	14.6	10.8	11.8	3.3
Transcription	6.3	7.0	6.1	5.9	5.1	7.9	0.0	8.5	7.6	6.1	7.6	3.1	6.8	2.6	4.9	4.1	5.6	2.3
Translation and protein synthesis	26.6	41.9	27.0	25.4	29.1	33.3	36.4	28.7	25.3	31.8	24.1	24.6	33.9	23.7	24.4	27.0	28.9	5.2
Transport	11.4	4.7	14.8	16.1	11.4	12.7	9.1	11.7	13.9	9.1	12.7	10.8	6.8	13.2	7.3	9.5	10.9	3.1
Uncharacterized/Unknown	10.1	7.0	12.2	11.9	7.6	9.5	13.6	8.5	17.7	12.1	7.6	16.9	11.9	13.2	22.0	14.9	12.3	4.1

Mean (±S.D) of the relative percentage per treatment found at both experimental designs. The percentage of proteins found to be down- or upregulated with respect to the negative control (H) are highlighted in red and blue, respectively. Statherin (A), histatin 3 (B), DR9 (C), DR9–DR9 (D), DR9–RR14 (E), RR14 (F), fluoride (G), NaCl (H).

## Data Availability

Data are contained within the article or supplementary material. The data presented in this study are available in Supplementary Tables S1–S4.

## Supplementary Materials

The following are available online at https://www.mdpi.com/article/10.3390/microorganisms10040742/s1: Supplementary Table S1: List of cellular proteins identified when the proteins/peptides were used only to form the AEP (experimental condition 1). Supplementary Table S2: List of ECM proteins identified when the proteins/peptides were used only to form the AEP (experimental condition 1). Supplementary Table S3: List of cellular proteins identified when the proteins/peptides were used to form the AEP and as daily treatments (experimental condition 2). Supplementary Table S4: List of ECM proteins identified when the proteins/peptides were used to form the AEP and as daily treatments (experimental condition 2).
