# Supplementary material for: Engineered Salivary Peptides Reduce Enamel Demineralization Provoked by Cariogenic S. mutans Biofilm
Source: Microorganisms. 2022 Mar 30;10(4):742. doi: 10.3390/microorganisms10040742 (PMC9032980; doi:10.3390/microorganisms10040742)
Supplement: Supplementary file 1 [file microorganisms-10-00742-s001.zip › S1_Table.pdf]

Supplementary Table S1. List of cellular proteins identified when the proteins/peptides were used only to form the AEP (experimental condition 1).

Intracellular proteins											
Accession	Gene name	Protein name	Protein function	Treatment							
				A	B	C	D	E	F	G	H
Adaptative responses to environmental changes											
Q8DTJ8	bacD	Putative bacitracin synthetase	Antibiotic biosynthesis								x
Q8DWD4	adhD	Dihydrolipoyl dehydrogenase	Cell redox homeostasis	x							x
Q8DT76	addA	ATP-dependent helicase/nuclease subunit A	DNA repair				x	x			
Q8DRX0	mutL	DNA mismatch repair protein MutL	DNA repair								x
P27624	recA	Protein RecA	DNA repair	x			x		x		x
Q8DT81	SMU_1485c	Putative endonuclease	DNA repair			x					
Q8DRU4	SMU_2120c	Putative 3-methyladenine DNA glycosylase	DNA repair								x
Q8DWN4	trcF	Transcription-repair-coupling factor	DNA repair			x					
P72481	uvrA	UvrABC system protein A	DNA repair			x	x	x		x	
Q8CWX7	uvrB	UvrABC system protein B	DNA repair			x					
Q8DSP2	SMU_1733c	Putative SNF helicase	DNA replication, recombination and repair			x					
Q8DSU5	SMU_1664c	Putative acetoin utilization protein, acetoin dehydrogenase	Energy store								x
Q8DW04	SMU_287	Putative ComB, accessory factor for ComA	Quorum sensing			x	x				
Q8DSH1	scnK	Putative histidine kinase, ScnK-like protein	Signal transduction				x				
Amino acid metabolism and biosynthesis											
Q8DUV8	aroA	3-phosphoshikimate 1-carboxyvinyltransferase	Amino acid biosynthesis				x				
Q8DUV7	aroK	Shikimate kinase	Amino acid biosynthesis			x					
Q8DUP3	carB	Carbamoyl-phosphate synthase large chain	Amino acid biosynthesis	x		x					
Q8DVU9	glnA	Glutamine synthetase type 1 glutamate--ammonia ligase	Amino acid biosynthesis			x			x		x
Q8DW43	ilvC	Ketol-acid reductoisomerase (NADP(+))	Amino acid biosynthesis	x	x	x	x	x	x	x	x
Q8DTW7	ilvE	Branched-chain-amino-acid aminotransferase	Amino acid biosynthesis			x					

Q8DSV3	serC	Phosphoserine aminotransferase	Amino acid biosynthesis			x	x	x			
Q8DVY6	SMU_318	N-acetyldiaminopimelate deacetylase	Amino acid biosynthesis				x				
Q8DUR2	SMU_841	Putative aminotransferase	Amino acid biosynthesis	x		x					
Q8DUG5	SMU_965	Homoserine dehydrogenase	Amino acid biosynthesis			x					
Q8DVF8	trpE	Anthranilate synthase component 1	Amino acid biosynthesis				x		x		
Q8DUL2	SMU_913	Glutamate dehydrogenase	Amino acid metabolism					x			

Bacterial adherence and biofilm formation

Q54443	dexA	Dextranase	Adherence				x			x	
Q9KIJ3	sloC	Metal ABC transporter substrate-binding lipoprotein	Adherence					x			
P11000	wapA	Wall-associated protein	Adherence			x				x	x
Q8DU58	wapE	Uncharacterized protein	Adherence			x		x			x
Q8DVR0	brpA	Biofilm regulatory protein A	Biofilm formation			x		x			x
Q8DWM3	gbpB	Putative secreted antigen GbpB/SagA putative peptidoglycan hydrolase	Biofilm formation	x							
Q8DUW9	gbpD	Glucan-binding protein D with lipase activity BglB-like protein	Biofilm formation						x		
Q8CVC7	SMU_609	Putative 40K cell wall protein	Biofilm formation				x				
P23504	spaP	Cell surface antigen I/II	Cell wall antigen	x	x	x	x	x	x	x	x
P11701	ftf	Levansucrase	EPS biosynthesis	x			x				
P08987	gtfB	Glucosyltransferase-I	EPS biosynthesis			x					x
P13470	gtfC	Glucosyltransferase-SI	EPS biosynthesis	x		x	x	x			x
Q8DUS4	rgpA	Putative RgpAc glycosyltransferase	EPS biosynthesis	x			x		x		
Q8CVC4	SMU_1432c	Glucanase	EPS catabolism				x				

Carbohydrate metabolism and energy production

Q8DT55	phsG	Alpha-1,4 glucan phosphorylase	Carbohydrate metabolic process				x				
Q03174	fruA	Fructan beta-fructosidase	Carbohydrate metabolic process	x		x	x				x
Q8DWH3	fruB	Fructan hydrolase exo-beta-D-fructosidase FruB	Carbohydrate metabolic process			x					
Q8DWE8	fruP	Tagatose-6-phosphate kinase	Carbohydrate metabolic process				x				

Q8DVV3	gapC	Glyceraldehyde-3-phosphate dehydrogenase	Carbohydrate metabolic process	x	x	x	x	x	x	x	x
Q8DT31	glgP	Alpha-1,4 glucan phosphorylase	Carbohydrate metabolic process								x
Q59934	pfl	Formate acetyltransferase	Carbohydrate metabolic process	x	x	x	x	x	x		x
Q8CWY1	pfl2	Formate acetyltransferase (Pyruvate formate-lyase 2)	Carbohydrate metabolic process	x							
Q8DU72	pgm	Putative phosphoglucomutase	Carbohydrate metabolic process								x
P95780	rmlB	dTDP-glucose 4,6-dehydratase	Carbohydrate metabolic process	x			x	x			x
Q8DRZ0	SMU_2065	Putative UDP-glucose-4-epimerase	Carbohydrate metabolic process	x							
I6L8Z8	SMU_308	Sorbitol-6-phosphate 2-dehydrogenase	Carbohydrate metabolic process				x				
Q8DVN9	SMU_435	Putative N-acetylglucosamine-6-phosphate deacetylase	Carbohydrate metabolic process				x				
O68579	ppaC	Probable manganese-dependent inorganic pyrophosphatase	Energy metabolism	x	x	x	x	x	x	x	x
Q8DTS9	eno	Enolase	Glycolytic process	x	x	x	x	x	x	x	x
Q8DWG0	fbaA	Fructose-1,6-biphosphate aldolase	Glycolytic process	x	x	x	x	x	x	x	x
P26283	ldh	L-lactate dehydrogenase	Glycolytic process	x		x	x		x		x
Q8DVV2	pgk	Phosphoglycerate kinase	Glycolytic process	x	x	x	x	x	x	x	x
Q8DTX7	pykF	Pyruvate kinase	Glycolytic process	x	x	x	x	x	x	x	x
P72484	tpiA	Triosephosphate isomerase	Glycolytic process			x					

Cell division, replication and cell wall synthesis

Q8DVD4	divIVA	Putative cell division protein DivIVA	Cell division	x		x	x	x			x
Q8DTL0	ftsE	Cell division ATP-binding protein FtsE	Cell division					x	x		x
Q8DVD9	ftsZ	Cell division protein FtsZ	Cell division			x					
Q8DSP6	mltG	Endolytic murein transglycosylase	Cell wall biosynthesis			x					
Q8DVM5	pbp2x	Putative penicillin-binding protein 2X	Cell wall biosynthesis		x	x					
Q8DUJ1	SMU_937	Putative mevalonate diphosphate decarboxylase	Peptidoglycan biosynthesis				x				
Q8DT49	ligA	DNA ligase	Replication								x
Q8DRY2	nrdD	Putative anaerobic ribonucleoside-triphosphate reductase	Replication			x					
Q8DU41	gyrA	DNA gyrase subunit A	Replication								x

Nucleoside/Nucleotide metabolism and biosynthesis

Q8DRR2	guaB	Inosine-5'-monophosphate dehydrogenase	Nucleotide biosynthesis						x		
Q8DWJ5	purE	N5-carboxyaminoimidazole ribonucleotide mutase	Nucleotide biosynthesis		x			x			
Q8DWL3	purF	Amidophosphoribosyltransferase	Nucleotide biosynthesis				x		x		
Q8DWL5	purL	Phosphoribosylformylglycinamidine synthase	Nucleotide biosynthesis	x			x				
Q8DW83	SMU_187c	tRNA-dihydrouridine synthase	Nucleotide biosynthesis				x				
I6L8Y1	SMU_273	Putative hexulose-6-phosphate synthase	Nucleotide biosynthesis								
Q8DVL6	SMU_464	Nicotinate phosphoribosyltransferase	Nucleotide biosynthesis	x	x		x	x			x
Q8CVC5	SMU_1213c	Putative 5'-nucleotidase	Nucleotide catabolism				x				

Other metabolic processes

Q8DWB9	adhE	Aldehyde-alcohol dehydrogenase	Alcohol metabolic process	x							
Q59925	fhs	Formate--tetrahydrofolate ligase	Biomolecules metabolism		x						
Q8DV28	SMU_689	Lysozyme	Cell wall catabolism	x							
Q9XB21	hup	DNA-binding protein HU	Chromosome condensation	x	x	x	x	x	x	x	x
Q7ZAL0	smc	Chromosome partition protein Smc	Chromosome condensation	x							
Q8DVB5	xseA	Exodeoxyribonuclease 7 large subunit	DNA catabolism				x			x	
Q8DVI3	SMU_505	Methyltransferase	DNA methylation	x							
Q8DSN3	acp	acyl carrier protein	Fatty acid biosynthesis							x	
Q8DSN6	fabF	3-oxoacyl-[acyl-carrier-protein] synthase 2	Fatty acid biosynthesis					x			
P34001	SMU_988	Uncharacterized protein	Lipid byosinthesis							x	
Q93D83	gpsA	Glycerol-3-phosphate dehydrogenase [NAD(P)+]	Lipid metabolism			x	x				
Q8DS57	ackA	acetate kinase	Metabolic processes					x			
Q8DTJ5	bacA1	Putative bacitracin synthetase 1 BacA	Metabolic processes		x	x	x			x	
Q8DTJ4	SMU_1343c	Putative polyketide synthase	Metabolic processes					x			
Q8DW46	SMU_229	Uncharacterized protein	Metabolic processes						x		
Q8DVN8	SMU_438c	Putative (R)-2-hydroxyglutaryl-CoA dehydratase activator-related protein	Metabolic processes								x
Q8DV33	SMU_683	Putative ATP-binding protein	Metabolic processes			x					

Q8DUJ0	SMU_938	Putative phosphomevalonate kinase	Metabolic processes					x			
Q8DVF0	dpr	Peroxide resistance protein Dpr	Oxidation-reduction process	x	x	x	x	x	x	x	x
Q8DTN9	flaW	Putative flavodoxin	Oxidation-reduction process								x
Q59931	gapN	NADP-dependent glyceraldehyde-3-phosphate dehydrogenase	Oxidation-reduction process	x	x	x	x	x	x	x	x
Q8DVU1	SMU_374	Putative oxidoreductase	Oxidation-reduction process					x			
Q8DWM7	ftsH	ATP-dependent zinc metalloprotease FtsH	Proteolysis			x	x	x	x		x
Q93D93	htpX	Protease HtpX homolog	Proteolysis								x
Q8DRQ6	htrA	Serine protease HtrA	Proteolysis				x	x			
Q8DS80	pbp2a	Putative membrane carboxypeptidase, penicillin-binding protein 2a	Proteolysis	x		x					
Q8DU26	pepN	aminopeptidase	Proteolysis					x			
Q8DSE4	pepP	Putative aminopeptidase P	Proteolysis			x					
Q8DVS2	pepX	Xaa-Pro dipeptidyl-peptidase	Proteolysis					x			
Q8DRR6	SMU_2153c	Putative peptidase	Proteolysis					x			
Q8DWB2	pnp	Polyribonucleotide nucleotidyltransferase	RNA degradation				x	x			x
Q8DUB2	SMU_1030	Putative polyribonucleotide nucleotidyltransferase Tn916 ORF8-like	RNA degradation	x							x
Q8DSQ0	SMU_1724c	Putative rRNA methylase	RNA methylation					x			
Q8DVG8	rlmN	Probable dual-specificity RNA methyltransferase RlmN	RNA methylation	x							
Q8DV10	ppc	Phosphoenolpyruvate carboxylase	Tricarboxylic acid cycle	x		x	x				

Transcription

Q8DUH3	clp	Putative Clp-like ATP-dependent protease, ATP-binding subunit	Transcription	x	x	x	x	x	x		x
Q8DUN0	galR	Galactose operon repressor GalR	Transcription			x					x
Q8DSP7	greA	Transcription elongation factor GreA	Transcription	x	x	x	x	x	x		x
Q02425	mtlR	Putative transcriptional regulator MtlR	Transcription				x				
I6L8Z6	psaR	Putative transcriptional regulator	Transcription								x
Q8DVV6	purR	Purine operon represso	Transcription			x					
Q8DS36	rpoA	DNA-directed RNA polymerase subunit alpha	Transcription				x				
Q8DS46	rpoB	DNA-directed RNA polymerase subunit beta(rpoB)	Transcription	x					x		

Q8DS47	rpoC	DNA-directed RNA polymerase subunit beta'	Transcription	x	x	x	x	x	x		x
Q8DWE9	SMU_112c	Putative transcriptional regulator	Transcription			x					
Q8DWD9	SMU_124	Putative transcriptional regulator (MarR family)	Transcription			x		x			x
Q8DSJ4	SMU_1789c	Probable transcriptional regulatory protein SMU_1789c	Transcription	x					x		
Q8DW49	SMU_226c	Putative transposase	Transcription				x				
Q8DV66	SMU_640c	Putative transcriptional regulator (GntR family)	Transcription								x
Q8DUH6	SMU_953c	Putative transcriptional regulator/aminotransferase	Transcription								x
Q8DVK3	sunL	Putative RNA-binding Sun protein possible rRNA methylase	Transcription				x				
Translation and protein synthesis											
Q8CWY0	alaS	Alanine--tRNA ligase	Protein biosynthesis			x	x				x
Q8DVD3	ileS	Isoleucine--tRNA ligase	Protein biosynthesis			x					
Q8CWX2	pheT	Phenylalanine--tRNA ligase beta subunit	Protein biosynthesis			x					
Q8DWH2	dnaJ	Chaperone protein DnaJ	Protein folding		x	x					x
O06942	dnaK	Chaperone protein DnaK	Protein folding	x	x	x	x	x	x	x	x
Q8CWW6	groL	60 kDa chaperonin	Protein folding	x	x	x	x	x	x	x	x
Q8CWW5	groS	10 kDa chaperonin	Protein folding	x	x	x	x			x	x
O06941	grpE	Protein GrpE	Protein folding			x	x		x		x
Q8CWZ3	hslO	33 kDa chaperonin	Protein folding				x				
Q8CVC6	prsA	Foldase protein PrsA	Protein folding			x					
Q8CWZ6	tig	Trigger factor	Protein folding	x	x	x	x	x	x	x	x
Q54431	ffh	Signal recognition particle protein	Protein targeting						x		
Q8DVV4	fusA	Elongation factor G	Translation	x	x	x	x	x	x		x
Q8DSG5	gatA	Glutamyl-tRNA(Gln) amidotransferase subunit A	Translation				x				
Q8DSG6	gatB	Aspartyl/glutamyl-tRNA(Asn/Gln) amidotransferase subunit B	Translation	x		x	x		x		x
Q8DVI8	hpf	Ribosome hibernation promoting factor	Translation	x		x		x			x
Q8DVP9	infB	Translation initiation factor IF-2	Translation	x			x	x	x		x
Q8DTF3	lepA	Elongation factor 4	Translation				x				

Q8DRS4	mnmA	tRNA-specific 2-thiouridylase MnmA	Translation				x	x			
Q8DSY0	rplA	50S ribosomal protein L1	Translation								x
G1XVB9	rplB	50S ribosomal protein L2	Translation			x			x		
Q8DS17	rplD	50S ribosomal protein L4	Translation			x				x	x
Q8DUH2	rplJ	50S ribosomal protein L10	Translation			x		x			
Q8DUG9	rplL	50S ribosomal protein L7/L12	Translation	x	x	x	x	x	x	x	x
Q8DS23	rplN	50S ribosomal protein L14	Translation	x		x	x	x	x		x
Q8DS31	rplO	50S ribosomal protein L15	Translation				x				
Q8DS20	rplP	50S ribosomal protein L16	Translation				x	x			x
Q8DS37	rplQ	50S ribosomal protein L17	Translation		x	x		x	x	x	x
Q8DS29	rplR	50S ribosomal protein L18	Translation			x		x		x	
Q8DUQ6	rplU	50S ribosomal protein L21	Translation			x	x	x		x	
Q8DS19	rplV	50S ribosomal protein L22	Translation	x	x		x	x	x		x
Q8DS24	rplX	50S ribosomal protein L24	Translation	x	x	x	x	x		x	x
Q8DS11	rpsB	30S ribosomal protein S2	Translation	x	x	x	x	x	x		x
P59186	rpsC	30S ribosomal protein S3	Translation			x	x	x		x	
P59133	rpsD	30S ribosomal protein S4	Translation	x	x	x	x	x	x		x
P59125	rpsE	30S ribosomal protein S5	Translation		x		x	x	x	x	x
Q8DSD7	rpsF	30S ribosomal protein S6	Translation	x		x	x				x
Q8DVV5	rpsG	30S ribosomal protein S7	Translation	x							
Q8DW97	rpsI	30S ribosomal protein S9	Translation	x	x	x	x	x	x		x
Q8DS35	rpsM	30S ribosomal protein S13	Translation	x	x				x	x	
P66474	rpsR	30S ribosomal protein S18	Translation		x						
Q8DTW9	rs1	Putative ribosomal protein S1 sequence specific DNA-binding protein	Translation	x		x	x	x	x	x	x
Q8DTF9	SMU_1388	Putative RNA helicase	Translation			x					
Q8DWH1	truA	tRNA pseudouridine synthase A	Translation						x		
Q8DS12	tsf	Elongation factor Ts	Translation	x	x	x	x			x	x
P72483	tuf	Elongation factor Tu	Translation	x	x	x	x	x	x	x	x

Q8CWW9	vacB	Ribonuclease R	Translation				x				
Transport											
Q8DW36	SMU_241c	Putative ABC transporter, ATP-binding protein amino acid transport system	Amino acid transport				x				
Q8DUY4	SMU_746c	Putative two-component membrane permease complex subunit	Amino acid transport			x					
Q8DUT1	SMU_815	Putative amino acid transporter, amino acid-binding protein	Amino acid transport				x			x	
Q8DW22	oppD	Putative oligopeptide ABC transporter, ATP-binding protein OppD	Peptide transport	x							
Q8DU24	pstB1	Phosphate import ATP-binding protein PstB 1	Phosphate transport								x
Q8DSF0	secA	Protein translocase subunit SecA	Protein transport	x							x
Q8DUN3	fruI	Inducible fructose permease	Sugar transport			x		x	x		x
Q8DT27	malF	Putative maltose/maltodextrin ABC transporter, permease protein MalF	Sugar transport				x				
Q8DS05	ptsG	Putative PTS system, glucose-specific IIABC component	Sugar transport			x		x			x
P45595	ptsI	Phosphoenolpyruvate-protein phosphotransferase	Sugar transport	x		x	x	x	x	x	x
Q8DU37	SMU_1120	Putative sugar ABC transporter, ATP-binding protein	Sugar transport				x				
Q8DSC2	SMU_1879	Putative PTS system, mannose-specific component IID	Sugar transport				x	x	x		x
Q8DS76	SMU_1957	Putative PTS system, mannose-specific IID component	Sugar transport					x			x
Q8DS75	SMU_1958c	Putative PTS system, mannose-specific IIC component	Sugar transport		x			x			
Q8DS74	SMU_1960c	Putative PTS system, mannose-specific IIB component	Sugar transport	x		x	x	x	x	x	x
Q8DS73	SMU_1961c	Putative PTS system, sugar-specific enzyme IIA component	Sugar transport						x	x	
P72477	abcX	Putative ABC transporter ATP-binding protein	Transport				x				
Q8DSU0	livK	Putative ABC transporter, branched chain amino acid-binding protein	Transport						x		
Q8DW25	oppA	Putative oligopeptide ABC transporter, substrate-binding protein OppA	Transport			x					
Q8DU85	opuAb	Putative ABC transporter, proline/glycine betaine permease protein	Transport	x							
Q8DRU6	opuCc	Putative ABC transporter osmoprotectant-binding protein, glycine betaine/carnitine/choline ABC transporter	Transport	x							
Q8DT32	pacL	Putative cation-transporting P-type ATPase PacL	Transport			x	x				
Q8DU70	SMU_1079c	Putative ABC transporter, ATP-binding protein	Transport			x					
Q8DU57	SMU_1093	Putative ABC transporter, permease protein	Transport	x			x		x		x

Q8DTZ3	SMU_1167c	Putative ABC transporter, ATP-binding protein	Transport			x	x				
Q8DTV6	SMU_1217c	Putative ABC transporter amino acid binding protein	Transport				x				
Q8DTP4	SMU_1289c	Putative permease, chloride channel	Transport				x				
Q8DTD8	SMU_1412c	Putative ABC transporter, membrane protein subunit and ATP-binding protein	Transport	x		x	x	x			
Q8DT43	SMU_1551c	Putative ABC transporter, ATP-binding protein	Transport			x					
Q8DSZ6	SMU_1605	Putative MDR permease possible transmembrane efflux protein	Transport			x					
Q8DSJ6	SMU_1787c	Putative secreted protein	Transport			x		x	x		
Q8DW35	SMU_242c	Putative amino acid ABC transporter, permease protein, glutamine transport system	Transport								x
Q8DW32	SMU_247	Putative ABC transporter ATP-binding protein	Transport	x	x	x					x
Q8DW31	SMU_248	Putative ABC transporter, membrane protein	Transport				x				
Q8DW05	SMU_286	Putative ABC transporter ATP-binding protein ComA	Transport			x					
Q8DVG6	SMU_524	Putative ABC transporter, ATP-binding protein	Transport			x					
Q8DUT7	SMU_806c	Putative glutamine ABC transporter, permease protein	Transport				x				
Q8DUL7	SMU_902	Putative ABC transporter, ATP-binding protein	Transport				x				
Q8DUK5	SMU_922	Putative ABC transporter, ATP-binding protein	Transport				x				
Q8DUK4	SMU_923	Putative ABC transporter, ATP-binding protein	Transport				x				
Q8DUJ3	SMU_935	Putative amino acid ABC transporter, permease protein	Transport			x					
Uncharacterized/Unknown											
Q8CWZ5	SMU_109	Uncharacterized protein	Uncharacterized					x			
Q8DU49	SMU_1104c	Uncharacterized protein	Uncharacterized			x					
Q8DWE4	SMU_117c	Uncharacterized protein	Uncharacterized				x				
Q8DWC3	SMU_141	Uncharacterized protein	Uncharacterized				x				
Q8DTC5	SMU_1427c	Uncharacterized protein	Uncharacterized								x
Q8DTB0	SMU_1447c	Uncharacterized protein	Uncharacterized				x			x	x
Q8DT86	SMU_1479	Uncharacterized protein	Uncharacterized	x	x	x	x	x	x		x
Q8DT20	SMU_1576c	Uncharacterized protein	Uncharacterized			x					
Q8DT19	SMU_1577c	Uncharacterized protein	Uncharacterized						x		

Q8DSS1	SMU_1700c	Uncharacterized protein	Uncharacterized								x
Q8DSM0	SMU_1760c	Uncharacterized protein	Uncharacterized	x		x	x				
Q8DSF8	SMU_1830c	Uncharacterized protein	Uncharacterized	x						x	
Q8DSA4	SMU_1904c	Uncharacterized protein	Uncharacterized			x	x				
Q8DRZ2	SMU_2061	Uncharacterized protein	Uncharacterized							x	
Q8DW41	SMU_235	Uncharacterized protein	Uncharacterized	x	x	x	x	x	x	x	x
Q8DVY0	SMU_329	Uncharacterized protein	Uncharacterized			x					
Q8DVX6	SMU_333	Uncharacterized protein	Uncharacterized	x							
Q8DVW2	SMU_350	Uncharacterized protein	Uncharacterized			x					
Q8DVJ1	SMU_497c	Uncharacterized protein	Uncharacterized				x				
Q8DVI6	SMU_502	Uncharacterized protein	Uncharacterized	x			x		x		
Q8DVH1	SMU_518	Uncharacterized protein	Uncharacterized				x				
Q8DV76	SMU_630	Uncharacterized protein	Uncharacterized			x					
Q8DWI5	SMU_63c	Uncharacterized protein	Uncharacterized	x		x	x	x	x		x
Q8DV34	SMU_682	Uncharacterized protein	Uncharacterized			x					
Q8DV27	SMU_690	Uncharacterized protein	Uncharacterized			x					
Q8DUY9	SMU_739c	Uncharacterized protein	Uncharacterized		x						
Q8DUR8	SMU_835	Uncharacterized protein	Uncharacterized				x				
Q8DUN2	SMU_874	Uncharacterized protein	Uncharacterized					x			
Q8DUH1	SMU_958	Uncharacterized protein	Uncharacterized						x		
Q8DTJ0	SMU_1347c	Uncharacterized protein	Unknown				x	x			
Q8DRT3	SMU_2133c	Putative membrane protein	Unknown	x		x	x			x	x
Q8DUV9	SMU_782	UPF0342 protein	Unknown			x				x	x
