# Supplementary material for: Engineered Salivary Peptides Reduce Enamel Demineralization Provoked by Cariogenic S. mutans Biofilm
Source: Microorganisms. 2022 Mar 30;10(4):742. doi: 10.3390/microorganisms10040742 (PMC9032980; doi:10.3390/microorganisms10040742)
Supplement: Supplementary file 1 [file microorganisms-10-00742-s001.zip › S2_Table.pdf]

Supplementary Table S2. List of ECM proteins identified when the proteins/peptides were used only to form the AEP (experimental condition 1).

ECM proteins											
Accession	Gene name	Protein name	Protein function	Treatment							
				A	B	C	D	E	F	G	H
Adaptative responses to environmental changes											
Q8DW47	SMU_228	Putative alkaline-shock protein-like protein	Alkaline shock	x			x	x		x	
Q8DTJ8	bacD	Putative bacitracin synthetase	Antibiotic biosynthesis				x				x
Q8DWD4	adhD	Dihydrolipoyl dehydrogenase	Cell redox homeostasis	x		x	x	x		x	
I6L8Y0	ahpC	alkyl hydroperoxide reductase	Cell redox homeostasis					x			
I6L922	SMU_765	NADH oxidase/alkyl hydroperoxidase reductase peroxide-forming	Cell redox homeostasis		x						
Q8DUK3	tpx	Thiol peroxidase	Cell redox homeostasis	x	x	x	x	x	x	x	x
Q8DSD2	trxA	Thioredoxin	Cell redox homeostasis	x	x	x	x	x	x	x	x
P09738	sodA	Superoxide dismutase [Mn/Fe]	Cellular detoxification	x	x	x	x	x	x	x	x
Q03JI6	cas9-2	CRISPR-associated endonuclease Cas9 2	CRISP element metabolism		x						
Q8DUM6	hsdS	Putative type I restriction-modification system, specificity determinant	DNA modification				x		x		
Q8DVY2	radA	DNA repair protein RadA	DNA repair					x			
P27624	recA	Protein RecA	DNA repair	x	x	x	x	x	x	x	x
Q9AIV4	smnA	Nuclease SmnA	DNA repair		x						x
Q8DWN4	trcF	Transcription-repair-coupling factor	DNA repair		x	x		x			
Q8CWX7	uvrB	UvrABC system protein B	DNA repair					x			
Q8DSD8	ssb	Single-stranded DNA-binding protein	DNA replication, recombination and repair	x		x		x		x	
Q8DS68	ssb2	Single-stranded DNA-binding protein	DNA replication, recombination and repair							x	
Q8DUM2	SMU_897	Type I restriction enzyme R Protein	DNA restriction				x				
Q8DRX2	cinA	Putative competence-damage inducible protein	DNA transformation							x	
Q8DUD4	smf	Putative DNA processing Smf protein	DNA transformation							x	
Q8DSU5	SMU_1664c	Putative acetoin utilization protein, acetoin dehydrogenase	Energy store	x	x	x	x	x		x	
Q8DSJ5	SMU_1788c	Putative bacterocin transport accessory protein, Bta	Killing factor	x	x	x	x	x	x	x	x

Q8DW04	SMU_287	Putative ComB, accessory factor for ComA	Quorum sensing			x		x	x		
Q8DW76	SMU_198c	Putative conjugative transposon protein	Recombination	x	x	x				x	
Q8DVL4	pbp1a	Penicillin-binding protein 1a membrane carboxypeptidase	Response to antibiotics	x	x		x	x			
Q8DW95	SMU_173	Putative ppGpp-regulated growth inhibitor	Stress		x		x	x			x
Q8DSF9	SMU_1828	Universal stress protein	Stress	x	x	x	x	x	x		x
Amino acid metabolism and biosynthesis											
Q8DSM8	akh	Aspartokinase	Amino acid biosynthesis					x			
P59311	argC	N-acetyl-gamma-glutamyl-phosphate reductase	Amino acid biosynthesis			x					
Q8DUV8	aroA	3-phosphoshikimate 1-carboxyvinyltransferase	Amino acid biosynthesis	x							
Q8DUW4	aroD	3-dehydroquinate dehydratase	Amino acid biosynthesis	x	x		x				
Q8DUW3	aroE	Shikimate dehydrogenase (NADP(+))	Amino acid biosynthesis	x	x	x	x	x	x	x	x
Q8DSF1	aroH	Phospho-2-dehydro-3-deoxyheptonate aldolase	Amino acid biosynthesis	x			x	x	x		x
Q8DUV7	aroK	Shikimate kinase	Amino acid biosynthesis				x	x	x	x	x
P10539	asd	aspartate-semialdehyde dehydrogenase	Amino acid biosynthesis		x			x		x	
Q8DTM1	aspB	Asparagine--oxo-acid transaminase	Amino acid biosynthesis				x			x	
Q8DUP3	carB	Carbamoyl-phosphate synthase large chain	Amino acid biosynthesis				x				x
Q8DVJ2	cysK	Cysteine synthase	Amino acid biosynthesis	x							x
Q8DUE5	dapA	4-hydroxy-tetrahydrodipicolinate synthase	Amino acid biosynthesis	x							
Q8DUL9	dapB	4-hydroxy-tetrahydrodipicolinate reductase	Amino acid biosynthesis	x	x		x		x	x	
Q8DVY7	dapH	2,3,4,5-tetrahydropyridine-2,6-dicarboxylate N-acetyltransferase	Amino acid biosynthesis	x		x	x	x			x
Q8DVU9	glnA	Glutamine synthetase type 1 glutamate--ammonia ligase	Amino acid biosynthesis	x	x	x	x	x	x	x	x
Q8DU67	glyA	Serine hydroxymethyltransferase	Amino acid biosynthesis	x			x	x		x	
Q8DTQ9	hisB	Imidazoleglycerol-phosphate dehydratase	Amino acid biosynthesis		x						
Q8DTQ6	hisG	ATP phosphoribosyltransferase	Amino acid biosynthesis				x				
Q8DW42	ilvA	L-threonine dehydratase	Amino acid biosynthesis	x	x		x		x	x	x
Q8DW45	ilvB	Acetolactate synthase	Amino acid biosynthesis	x	x	x	x		x	x	x
Q8DW43	ilvC	Ketol-acid reductoisomerase (NADP(+))	Amino acid biosynthesis	x	x	x	x	x	x	x	x
Q8DRT7	ilvD	Dihydroxy-acid dehydratase	Amino acid biosynthesis	x	x		x		x	x	

Q8DTW7	ilvE	Branched-chain-amino-acid aminotransferase	Amino acid biosynthesis	x	x	x	x	x	x	x	x
Q8DTG2	leuA	2-isopropylmalate synthase	Amino acid biosynthesis	x	x	x	x	x	x		x
Q8DVM9	proA	Gamma-glutamyl phosphate reductase	Amino acid biosynthesis	x							
Q8DSV3	serC	Phosphoserine aminotransferase	Amino acid biosynthesis	x	x	x	x	x	x	x	x
Q8DUG5	SMU_965	Homoserine dehydrogenase	Amino acid biosynthesis		x	x	x	x	x		
Q8DVF4	trpF	N-(5'-phosphoribosyl)anthranilate isomerase	Amino acid biosynthesis						x		
Q8DUL2	SMU_913	Glutamate dehydrogenase	Amino acid metabolism	x	x	x	x	x	x	x	x
Q8DVD8	ylmE	Pyridoxal phosphate homeostasis protein	Amino acid metabolism					x			
Bacterial adherence and biofilm formation											
Q54443	dexA	Dextranase	Adherence	x	x	x	x	x	x	x	x
Q8DRV2	gbpA	Glucan-binding protein A, GbpA	Adherence	x	x	x	x	x	x	x	x
Q9KIJ3	sloC	Metal ABC transporter substrate-binding lipoprotein	Adherence	x	x		x	x	x	x	x
P11000	wapA	Wall-associated protein	Adherence	x	x	x	x		x	x	x
Q8DVR0	brpA	Biofilm regulatory protein A	Biofilm formation	x							
Q8DWM3	gbpB	Putative secreted antigen GbpB/SagA putative peptidoglycan hydrolase	Biofilm formation	x	x	x	x	x	x	x	x
Q8DTF1	gbpC	Glucan-binding protein C, GbpC	Biofilm formation		x		x	x	x	x	
Q8DUW9	gbpD	Glucan-binding protein D with lipase activity BglB-like protein	Biofilm formation						x		
Q8CVC7	SMU_609	Putative 40K cell wall protein	Biofilm formation	x	x	x	x	x	x	x	x
P23504	spaP	Cell surface antigen I/II	Cell wall antigen	x	x	x	x	x	x	x	x
P11701	ftf	Levansucrase	EPS biosynthesis	x	x	x	x	x	x	x	x
P08987	gtfB	Glucosyltransferase-I	EPS biosynthesis		x	x	x	x	x	x	x
P13470	gtfC	Glucosyltransferase-SI	EPS biosynthesis	x	x	x		x	x	x	x
P49331	gtfD	Glucosyltransferase-S	EPS biosynthesis	x	x	x	x	x	x	x	x
Q8DT30	malQ	4-alpha-glucanotransferase	EPS biosynthesis		x			x			
Carbohydrate metabolism and energy production											
Q8DT55	phsG	Alpha-1,4 glucan phosphorylase	Carbohydrate metabolic process				x				
P95778	rmlA	Glucose-1-phosphate thymidylyltransferase	Carbohydrate biosynthesis	x			x	x	x		x

Q8DUS5	SMU_824	dTDP-4-dehydrorhamnose reductase	Carbohydrate biosynthesis	x	x		x	x	x	x	x
Q8DTT9	rpiA	Ribose-5-phosphate isomerase A	Carbohydrate degradation	x	x		x	x	x	x	
Q8DT00	bgl	Putative phospho-beta-glucosidase	Carbohydrate metabolic process							x	
Q03174	fruA	Fructan beta-fructosidase	Carbohydrate metabolic process	x	x	x	x	x	x	x	x
P96994	galT	Galactose-1-phosphate uridylyltransferase	Carbohydrate metabolic process								x
Q8DVV3	gapC	Glyceraldehyde-3-phosphate dehydrogenase	Carbohydrate metabolic process	x	x	x	x	x	x	x	x
Q8DT31	glgP	Alpha-1,4 glucan phosphorylase	Carbohydrate metabolic process	x	x	x	x	x	x	x	x
Q8DTC6	glmM	Phosphoglucosamine mutase	Carbohydrate metabolic process	x	x	x	x	x		x	
Q8DTY0	glmS	Glutamine--fructose-6-phosphate aminotransferase [isomerizing]	Carbohydrate metabolic process	x	x			x	x	x	
Q8DV70	nagB	Glucosamine-6-phosphate deaminase	Carbohydrate metabolic process	x			x		x	x	
Q59934	pfl	Formate acetyltransferase	Carbohydrate metabolic process	x	x	x	x	x	x	x	x
Q8CWY1	pfl2	Formate acetyltransferase (Pyruvate formate-lyase 2)	Carbohydrate metabolic process	x	x	x	x	x		x	x
Q8DU72	pgm	Putative phosphoglucomutase	Carbohydrate metabolic process	x	x	x	x	x	x	x	x
P95780	rmlB	dTDP-glucose 4,6-dehydratase	Carbohydrate metabolic process	x						x	x
Q07211	scrK	Fructokinase	Carbohydrate metabolic process			x					
I6L930	SMU_322c	UTP--glucose-1-phosphate uridylyltransferase	Carbohydrate metabolic process	x							
O68579	ppaC	Probable manganese-dependent inorganic pyrophosphatase	Energy metabolism	x	x	x	x	x	x	x	x
Q8CWX0	glgA	Glycogen synthase	Glycogen biosynthesis				x				
Q8DTS9	eno	Enolase	Glycolytic process	x	x	x	x	x	x	x	x
Q8DWG0	fbaA	Fructose-1,6-biphosphate aldolase	Glycolytic process	x	x	x	x	x	x	x	x
Q8DVE8	glk	Putative glucose kinase	Glycolytic process				x	x			
P59161	gpmA	2,3-bisphosphoglycerate-dependent phosphoglycerate mutase	Glycolytic process	x	x	x	x	x	x	x	x
P26283	ldh	L-lactate dehydrogenase	Glycolytic process	x	x	x	x	x	x	x	x
Q8DTX6	pfkA	ATP-dependent 6-phosphofructokinase	Glycolytic process	x	x	x	x	x	x	x	x
Q9X670	pgi	Glucose-6-phosphate isomerase	Glycolytic process	x	x	x	x	x	x	x	x
Q8DVV2	pgk	Phosphoglycerate kinase	Glycolytic process	x	x	x	x	x	x	x	x
Q8DTX7	pykF	Pyruvate kinase	Glycolytic process	x	x	x	x	x	x	x	x
P72484	tpiA	Triosephosphate isomerase	Glycolytic process	x	x	x	x	x	x	x	x

Cell division, replication and cell wall synthesis

Q8DVL1	gpsB	Cell cycle protein GpsB	Cell cycle	x	x		x				
Q8DUU2	obg	GTPase Obg	Cell cycle					x			x
Q8DVD4	divIVA	Putative cell division protein DivIVA	Cell division	x	x	x	x	x	x	x	x
Q8DTQ3	ezrA	Septation ring formation regulator EzrA	Cell division							x	
Q8DVE0	ftsA	Cell division protein FtsA O	Cell division	x	x	x	x	x	x	x	x
Q8DTL0	ftsE	Cell division ATP-binding protein FtsE	Cell division					x			
Q8DVD9	ftsZ	Cell division protein FtsZ	Cell division	x	x	x	x	x	x	x	x
Q8DVD7	sepF	Cell division protein SepF	Cell division				x				
Q8DUE4	SMU_991	Putative ribonucleotide reductase	Cell division	x		x	x	x	x		x
Q8DTM9	whiA	Probable cell division protein WhiA	Cell division	x		x			x		
Q8DWM4	mreC	Cell shape-determining protein MreC	Cell shape	x							
Q8DSP6	mltG	Endolytic murein transglycosylase	Cell wall biosynthesis		x	x				x	
O70055	dltC	D-alanyl carrier protein	Cell wall biosynthesis		x	x		x			
Q8DV08	murN	Putative peptidoglycan branched peptide synthesis protein alanine adding enzyme beta-lactam resistance factor MurN	Cell wall biosynthesis							x	
Q8DVM5	pbp2x	Putative penicillin-binding protein 2X	Cell wall biosynthesis			x	x				
Q8DSP4	murC	UDP-N-acetylmuramate--L-alanine ligase	Cell wall biosynthesis							x	
Q8DVA0	pbp2b	Penicillin-binding protein 2b	Cell wall biosynthesis			x				x	
Q8DUM9	pbpX	Putative penicillin-binding protein, class C fmt-like protein	Cell wall biosynthesis						x		
Q8DT57	murA1	UDP-N-acetylglucosamine 1-carboxyvinyltransferase 1	Peptidoglycan biosynthesis				x				
Q8DT24	murA2	UDP-N-acetylglucosamine 1-carboxyvinyltransferase 2	Peptidoglycan biosynthesis	x				x	x		
Q8DST2	murE	UDP-N-acetylmuramoyl-L-alanyl-D-glutamate--L-lysine ligase	Peptidoglycan biosynthesis			x	x				
Q8DSQ5	murI	Glutamate racemase	Peptidoglycan biosynthesis								
Q8DV07	murM	Putative peptidoglycan branched peptide synthesis protein MurM	Peptidoglycan biosynthesis		x		x		x	x	
Q8DTM0	dinG	3'-5' exonuclease DinG	Replication			x					
Q8DWN8	dnaN	Beta sliding clamp	Replication					x	x		
Q8DT49	ligA	DNA ligase	Replication								
Q8DSK4	nrdI	Putative ribonucleotide reductase protein, Nrd	Replication						x		
Q8DV42	SMU_668c	Ribonucleoside-diphosphate reductase	Replication								x

Q8DWN9	dnaA	Chromosomal replication initiator protein DnaA	Replication		x		x		x		
Q8DU41	gyrA	DNA gyrase subunit A	Replication	x	x	x	x	x	x	x	
Q8DTQ2	gyrB	DNA gyrase subunit B	Replication		x					x	
Q8DTW6	parC	DNA topoisomerase 4 subunit A	Replication	x					x	x	

Nucleoside/Nucleotide metabolism and biosynthesis

Q8DS33	adk	adenylate kinase	Nucleotide biosynthesis	x		x	x	x	x	x	x
P95787	atpA	ATP synthase subunit alpha	Nucleotide biosynthesis	x	x	x	x	x	x	x	x
P95789	atpD	ATP synthase subunit beta	Nucleotide biosynthesis	x	x	x	x	x	x	x	x
Q8DU81	guaA	GMP synthase [glutamine-hydrolyzing]	Nucleotide biosynthesis							x	
Q8DRR2	guaB	Inosine-5'-monophosphate dehydrogenase	Nucleotide biosynthesis	x	x	x	x	x	x	x	x
Q8DU33	pdp	Putative pyrimidine-nucleoside phosphorylase	Nucleotide biosynthesis								x
Q8DWM2	prs1	Ribose-phosphate pyrophosphokinase 1	Nucleotide biosynthesis				x				
Q8DU94	prs2	Putative ribose-phosphate pyrophosphokinase 2	Nucleotide biosynthesis			x					
Q8DW14	purA	Adenylosuccinate synthetase	Nucleotide biosynthesis		x						
P72478	purB	adenylosuccinate lyase	Nucleotide biosynthesis	x	x	x	x	x	x	x	x
Q8DWJ7	purD	Phosphoribosylamine--glycine ligase	Nucleotide biosynthesis	x							
Q8DWJ5	purE	N5-carboxyaminoimidazole ribonucleotide mutase	Nucleotide biosynthesis	x		x	x	x		x	x
Q8DWK8	purH	Bifunctional purine biosynthesis protein PurH	Nucleotide biosynthesis				x				
Q8DTV2	pyrE	Orotate phosphoribosyltransferase	Nucleotide biosynthesis	x			x	x	x	x	x
Q8DWG1	pyrG	CTP synthase	Nucleotide biosynthesis	x	x			x			
Q8DSY1	pyrH	Uridylate kinase	Nucleotide biosynthesis				x				
Q8DS79	SMU_1950	Pseudouridine synthase	Nucleotide biosynthesis	x							
Q8DVL6	SMU_464	Nicotinate phosphoribosyltransferase	Nucleotide biosynthesis	x	x	x	x	x	x	x	x
Q8DST6	upp	Uracil phosphoribosyltransferase	Nucleotide biosynthesis	x		x	x	x	x	x	
Q8CVC5	SMU_1213c	Putative 5'-nucleotidase	Nucleotide catabolism	x	x	x	x	x	x	x	x
Q8DV23	cmk	Cytidylate kinase	Nucleotide metabolic process	x							

Other metabolic processes

Q8DTA7	aldB	Alpha-acetolactate decarboxylase	Acid metabolism	x							
Q8DWB9	adhE	Aldehyde-alcohol dehydrogenase	Alcohol metabolic process	x	x	x	x	x	x	x	x
Q8DV28	SMU_689	Lysozyme	Cell wall catabolism	x	x	x	x	x	x	x	x
Q9XB21	hup	DNA-binding protein HU	Chromosome condensation	x	x	x	x	x	x	x	x
Q8DVB5	xseA	Exodeoxyribonuclease 7 large subunit	DNA catabolism		x						
Q8DSP0	accD	Acetyl-coenzyme A carboxylase carboxyl transferase subunit beta	Fatty acid biosynthesis							x	
Q8DSN3	acp	acyl carrier protein	Fatty acid biosynthesis	x	x	x	x	x	x	x	x
Q8DSN6	fabF	3-oxoacyl-[acyl-carrier-protein] synthase 2	Fatty acid biosynthesis	x	x	x	x	x	x	x	x
Q8DSN5	fabG	Putative 3-oxoacyl-acyl-carrier-protein reductase / 3-ketoacyl-acyl carrier protein reductase	Fatty acid biosynthesis	x							
Q8DSN0	fabM	Trans-2-decenoyl-[acyl-carrier-protein] isomerase	Fatty acid biosynthesis	x	x					x	
Q8DSN8	fabZ	3-hydroxyacyl-[acyl-carrier-protein] dehydratase FabZ	Fatty acid biosynthesis	x						x	
Q93D83	gpsA	Glycerol-3-phosphate dehydrogenase [NAD(P)+]	Lipid metabolism			x					
Q8DTJ2	SMU_1345c	Putative peptide synthetase	Metabolic processes	x	x	x			x	x	
Q8DS57	ackA	acetate kinase	Metabolic processes			x	x	x		x	
Q8DWD5	adhC	Dihydrolipoamide acetyltransferase component of pyruvate dehydrogenase complex	Metabolic processes							x	x
Q8DTJ5	bacA1	Putative bacitracin synthetase 1 BacA	Metabolic processes	x	x	x	x	x		x	
Q8DTJ7	bacA2	Putative surfactin synthetase	Metabolic processes			x	x				
Q8CVC3	cah	Putative carbonic anhydrase	Metabolic processes		x	x		x	x	x	x
Q8CWW8	fabD	Malonyl CoA-acyl carrier protein transacylase	Metabolic processes	x		x	x	x	x	x	
Q8DVC3	feoB	Ferrous iron transport protein B	Metabolic processes			x					
Q8DVK1	pknB	Putative serine/threonine protein kinase	Metabolic processes		x						
Q8DUA0	SMU_1043c	Putative phosphotransacetylase	Metabolic processes	x	x	x	x	x	x	x	x
Q8DTM7	SMU_1306c	Nucleotide-binding protein	Metabolic processes	x							
Q8DTK0	SMU_1337c	Putative alpha/beta superfamily hydrolase	Metabolic processes	x		x	x			x	x
Q8DTJ4	SMU_1343c	Putative polyketide synthase	Metabolic processes					x			
Q8DSP9	SMU_1725	Acylphosphatase	Metabolic processes				x				
Q8DSI4	SMU_1801c	Putative GTP-binding protein	Metabolic processes	x							
Q8DW46	SMU_229	Uncharacterized protein	Metabolic processes	x	x					x	
Q8DWM1	SMU_24	aminotransferase	Metabolic processes	x	x	x	x	x		x	x

Q8DW28	SMU_251	Uncharacterized protein	Metabolic processes					x		x	
Q8DVE5	SMU_546	Putative GTP-binding protein	Metabolic processes	x	x	x	x	x	x	x	x
Q8DUW6	SMU_775c	Uncharacterized protein	Metabolic processes					x			
Q8DW01	tkt	Transketolase	Metabolic processes	x		x			x		
Q8DSG1	yfbQ	Putative aminotransferase	Metabolic processes							x	
Q8DWD7	adhA	Putative acetoin dehydrogenase (TPP-dependent), E1 component alpha subunit	Oxidation-reduction process			x					
Q8DWD6	adhB	Putative acetoin dehydrogenase (TPP-dependent), E1 component beta subunit	Oxidation-reduction process	x				x			x
Q8DTL3	budC	Putative acetoin dehydrogenase	Oxidation-reduction process	x	x	x	x	x	x	x	
Q8DVF0	dpr	Peroxide resistance protein Dpr	Oxidation-reduction process	x	x	x	x	x	x	x	x
Q8DTN9	flaW	Putative flavodoxin	Oxidation-reduction process	x	x	x	x	x	x	x	x
Q59931	gapN	NADP-dependent glyceraldehyde-3-phosphate dehydrogenase	Oxidation-reduction process	x	x	x	x	x	x	x	x
Q8CWY9	gltA	Glutamate synthase (Large subunit)	Oxidation-reduction process						x		
O68574	hlyX	Putative hemolysin	Oxidation-reduction process	x			x		x		
Q8DUA3	MU_1040c	Putative oxidoreductase, short-chain dehydrogenase/reductase	Oxidation-reduction process	x							
Q8DSV6	serA	Putative D-3-phosphoglycerate dehydrogenase	Oxidation-reduction process	x	x		x			x	
Q8DU52	SMU_1098c	Putative oxidoreductase	Oxidation-reduction process			x					
Q8DTT3	SMU_1240c	Putative nitroreductase	Oxidation-reduction process	x			x		x		x
Q8DTM4	SMU_1309c	Putative glycerol dehydrogenase	Oxidation-reduction process					x			
Q8DSN4	SMU_1742c	Putative trans-2-enoyl-ACP reductase	Oxidation-reduction process	x	x	x	x	x	x	x	x
Q8DSD3	SMU_1867c	Putative alcohol dehydrogenase	Oxidation-reduction process				x				
Q8DRT8	SMU_2127	Putative succinate semialdehyde dehydrogenase	Oxidation-reduction process		x			x			
Q8DUN5	SMU_869	Ferredoxin--NADP reductase	Oxidation-reduction process			x					
Q8DST7	clpP	ATP-dependent Clp protease proteolytic subunit	Proteolysis	x			x	x	x	x	x
Q8DWM7	ftsH	ATP-dependent zinc metalloprotease FtsH	Proteolysis	x	x	x	x	x	x	x	x
Q8DRQ6	htrA	Serine protease HtrA	Proteolysis						x		
Q8DW33	mecA	Adapter protein MecA	Proteolysis				x				
Q8DS80	pbp2a	Putative membrane carboxypeptidase, penicillin-binding protein 2a	Proteolysis	x							
Q8DS62	pepA	Putative glutamyl-aminopeptidase endo-1,4-beta-glucanase	Proteolysis							x	
Q8DSE4	pepP	Putative aminopeptidase P	Proteolysis				x				

Q8DTB6	SMU_1438c	Putative Zn-dependent protease	Proteolysis		x	x		x			
Q8DVK2	SMU_483	Putative phosphoprotein phosphatase (PppL protein)	Protein metabolism	x			x	x			
Q8DV90	cshA	DEAD-box ATP-dependent RNA helicase CshA	RNA catabolic process	x	x	x	x	x	x	x	x
Q8DWB2	pnp	Polyribonucleotide nucleotidyltransferase	RNA degradation	x	x	x	x	x	x	x	x
Q59938	acn	Aconitate hydratase A	Tricarboxylic acid cycle				x	x	x		
Q8DV10	ppc	Phosphoenolpyruvate carboxylase	Tricarboxylic acid cycle	x	x	x	x	x	x	x	x
Transcription											
Q8DTB3	rnj	Ribonuclease J	RNA processing	x	x		x		x	x	x
Q8DT90	rnz	Ribonuclease Z	RNA processing								
Q8DSY7	ybeY	Endoribonuclease YbeY	RNA processing	x							
O07329	ccpA	Catabolite control protein A	Transcription	x	x	x	x	x	x	x	x
Q8DUH3	clp	Putative Clp-like ATP-dependent protease, ATP-binding subunit	Transcription	x	x	x	x	x	x	x	x
Q8DVD0	clpE	ATP-dependent protease ClpE	Transcription	x	x	x	x	x	x	x	x
Q8DUN0	galR	Galactose operon repressor GalR	Transcription	x			x				
I6L8Z3	gcrR	Response regulator GcrR for glucan-binding protein C	Transcription	x	x	x	x	x	x	x	x
Q8DSP7	greA	Transcription elongation factor GreA	Transcription	x	x	x	x	x	x	x	x
Q02425	mtlR	Putative transcriptional regulator MtlR	Transcription					x			
Q8DVQ2	nusA	Transcription termination/antitermination protein NusA	Transcription			x	x	x	x	x	
Q8DS82	nusG	Transcription termination/antitermination protein NusG	Transcription	x	x	x	x	x	x	x	x
I6L8Z6	psaR	Putative transcriptional regulator	Transcription				x				
P45596	ptsH	Phosphocarrier protein HPr	Transcription	x			x	x	x	x	x
Q8DVV6	purR	Purine operon represso	Transcription	x					x		
Q8DU91	rex	Redox-sensing transcriptional repressor Rex	Transcription				x				
Q8DS36	rpoA	DNA-directed RNA polymerase subunit alpha	Transcription	x	x	x	x	x	x	x	x
Q8DS46	rpoB	DNA-directed RNA polymerase subunit beta(rpoB)	Transcription	x	x	x	x	x	x	x	x
Q8DS47	rpoC	DNA-directed RNA polymerase subunit beta'	Transcription	x	x	x	x	x	x	x	x
Q8DWG2	rpoE	Probable DNA-directed RNA polymerase subunit delta	Transcription	x	x	x	x	x	x	x	x
O33662	sigA	RNA polymerase sigma factor SigA	Transcription	x		x	x				

Q8DU13	SMU_1146c	Putative response regulator	Transcription				x				
Q8DTP9	SMU_1282	Putative transcriptional regulator	Transcription			x					
Q8DTP6	SMU_1287	Putative transcriptional regulator	Transcription			x					
Q8DSJ4	SMU_1789c	Probable transcriptional regulatory protein SMU_1789c	Transcription								x
Q8DS71	SMU_1964c	Putative response regulator	Transcription	x			x				
Q8DS66	SMU_1969c	Putative transcriptional regulator	Transcription			x					
Q8DU17	spxA	Regulatory protein Spx	Transcription		x						

Translation and protein synthesis

Q8CWY0	alaS	Alanine--tRNA ligase	Protein biosynthesis	x	x		x	x		x	x
Q8DRW2	argS	Arginine--tRNA ligase	Protein biosynthesis	x	x	x	x	x	x	x	x
Q8DSG3	aspS1	Aspartate--tRNA(Asp/Asn) ligase	Protein biosynthesis					x	x		
Q8DRV9	aspS2	Aspartate--tRNA ligase 2	Protein biosynthesis		x					x	
Q8DS90	der	GTPase Der	Protein biosynthesis					x			
Q8DVX9	gltX	Glutamate--tRNA ligase	Protein biosynthesis	x			x				
Q8CWY5	glyS	Glycine--tRNA ligase beta subunit	Protein biosynthesis					x		x	
Q8DVD3	ileS	Isoleucine--tRNA ligase	Protein biosynthesis	x	x	x	x	x	x	x	x
Q8DS85	leuS	Leucine--tRNA ligase	Protein biosynthesis				x	x	x	x	
Q8CWX2	pheT	Phenylalanine--tRNA ligase beta subunit	Protein biosynthesis	x	x		x			x	x
Q8DSJ9	proS	Proline--tRNA ligase	Protein biosynthesis	x	x	x	x	x		x	x
Q8DTK9	rf2	Peptide chain release factor 2	Protein biosynthesis		x					x	
Q8DS21	rpmC	50S ribosomal protein L29	Protein biosynthesis	x	x	x	x				x
P59156	rsmA	Ribosomal RNA small subunit methyltransferase A	Protein biosynthesis					x			
Q8DS65	SMU_1970c	Phenylalanine--tRNA ligase beta subunit	Protein biosynthesis				x			x	
Q8DSL1	valS	Valine--tRNA ligase	Protein biosynthesis			x					
Q8DWN6	ychF	Ribosome-binding ATPase YchF	Protein biosynthesis							x	
Q8DS14	clpC	Class III stress response-related ATP-dependent Clp protease, ATP-binding subunit	Protein folding		x	x	x		x	x	x
Q8DUI0	clpX	ATP-dependent Clp protease ATP-binding subunit ClpX	Protein folding	x	x		x	x	x	x	
Q8DWH2	dnaJ	Chaperone protein DnaJ	Protein folding	x	x	x	x	x	x		x

O06942	dnaK	Chaperone protein DnaK	Protein folding	x	x	x	x	x	x	x	x
Q8CWW6	groL	60 kDa chaperonin	Protein folding	x	x	x	x	x	x	x	x
Q8CWW5	groS	10 kDa chaperonin	Protein folding	x	x	x	x	x	x	x	x
O06941	grpE	Protein GrpE	Protein folding	x	x	x	x	x	x	x	x
Q8CVC6	prsA	Foldase protein PrsA	Protein folding	x	x			x		x	x
Q8DVJ7	SMU_488	Putative hydrolase	Protein folding	x		x	x	x	x		x
Q8CWZ6	tig	Trigger factor	Protein folding	x	x	x	x	x	x	x	x
Q54431	ffh	Signal recognition particle protein	Protein targeting				x	x	x	x	x
Q8DTM5	aldR	Putative translation initiation inhibitor aldR regulator-like protein	Translation	x		x	x	x	x		x
Q8DVM2	cshB	DEAD-box ATP-dependent RNA helicase CshB	Translation		x				x		
Q8DWC2	def	Peptide deformylase	Translation	x	x	x	x	x	x		x
Q8DSE7	efp	Elongation factor P	Translation	x	x	x	x	x	x	x	x
Q8DSY2	frr	Ribosome-recycling factor	Translation	x	x	x	x	x	x	x	x
Q8DVV4	fusA	Elongation factor G	Translation	x	x	x	x	x	x	x	x
Q8DSG5	gatA	Glutamyl-tRNA(Gln) amidotransferase subunit A	Translation	x	x	x	x	x	x		x
Q8DSG6	gatB	Aspartyl/glutamyl-tRNA(Asn/Gln) amidotransferase subunit B	Translation	x	x		x	x	x	x	
Q8DSG4	gatC	Aspartyl/glutamyl-tRNA(Asn/Gln) amidotransferase subunit C	Translation	x	x	x	x	x	x	x	x
Q8DVI8	hpf	Ribosome hibernation promoting factor	Translation	x	x	x	x	x	x	x	x
Q8DS34	inf	Translation initiation factor IF-1	Translation	x		x			x		x
Q8DVP9	infB	Translation initiation factor IF-2	Translation	x	x	x	x	x	x	x	x
Q8DV22	infC	Translation initiation factor IF-3	Translation	x				x			
Q8DU64	prfA	Peptide chain release factor 1	Translation	x		x				x	x
Q8DVP8	rbfA	Ribosome-binding factor A	Translation	x							
Q8DSY0	rplA	50S ribosomal protein L1	Translation	x	x	x	x	x	x	x	x
G1XVB9	rplB	50S ribosomal protein L2	Translation	x	x	x	x	x	x	x	x
Q8DS16	rplC	50S ribosomal protein L3	Translation	x	x	x	x	x	x	x	x
Q8DS17	rplD	50S ribosomal protein L4	Translation	x	x	x	x	x	x	x	x
Q8DS25	rplE	50S ribosomal protein L5	Translation	x	x	x	x	x	x	x	x
Q8DS28	rplF	50S ribosomal protein L6	Translation	x	x	x	x	x	x	x	x

Q8DRS8	rplI	50S ribosomal protein L9	Translation	x	x	x	x	x	x	x	x
Q8DUH2	rplJ	50S ribosomal protein L10	Translation	x	x	x	x	x	x	x	x
Q8DSX9	rplK	50S ribosomal protein L11	Translation	x	x	x	x	x	x	x	x
Q8DUG9	rplL	50S ribosomal protein L7/L12	Translation	x	x	x	x	x	x	x	x
Q8DW98	rplM	50S ribosomal protein L13	Translation	x	x	x	x	x	x	x	x
Q8DS23	rplN	50S ribosomal protein L14	Translation	x	x	x	x	x	x	x	x
Q8DS31	rplO	50S ribosomal protein L15	Translation	x	x	x	x	x	x	x	x
Q8DS37	rplQ	50S ribosomal protein L17	Translation	x	x	x	x	x	x	x	x
Q8DS29	rplR	50S ribosomal protein L18	Translation	x	x	x	x	x	x	x	x
Q8DTP5	rplS	50S ribosomal protein L19	Translation				x			x	
Q8DV20	rplT	50S ribosomal protein L20	Translation	x			x			x	
Q8DUQ6	rplU	50S ribosomal protein L21	Translation	x	x	x	x	x	x	x	x
Q8DS19	rplV	50S ribosomal protein L22	Translation	x	x	x	x	x	x	x	x
G1XVC0	rplW	50S ribosomal protein L23	Translation	x	x	x	x	x	x	x	x
Q8DS24	rplX	50S ribosomal protein L24	Translation	x	x	x	x	x	x	x	x
Q8DUQ4	rpmA	50S ribosomal protein L27	Translation	x	x	x	x	x	x	x	x
Q8DS30	rpmD	50S ribosomal protein L30	Translation	x	x	x	x	x		x	
Q8DTN5	rpmE2	50S ribosomal protein L31 type B	Translation	x	x	x	x	x	x	x	x
Q8DRV7	rpmF	50S ribosomal protein L32	Translation							x	
Q8DS11	rpsB	30S ribosomal protein S2	Translation	x	x	x	x	x	x	x	x
P59186	rpsC	30S ribosomal protein S3	Translation	x	x	x	x	x	x	x	x
P59133	rpsD	30S ribosomal protein S4	Translation	x	x	x	x	x	x	x	x
P59125	rpsE	30S ribosomal protein S5	Translation	x	x	x	x	x	x	x	x
Q8DSD7	rpsF	30S ribosomal protein S6	Translation	x	x	x	x	x	x	x	x
Q8DVV5	rpsG	30S ribosomal protein S7	Translation	x	x	x	x	x	x	x	x
Q8DS27	rpsH	30S ribosomal protein S8(	Translation	x	x	x	x	x	x	x	x
Q8DW97	rpsI	30S ribosomal protein S9	Translation	x	x		x		x		x
P48853	rpsJ	30S ribosomal protein S10	Translation	x	x	x	x	x	x	x	x
P59378	rpsK	30S ribosomal protein S11	Translation	x	x	x	x	x	x	x	x

P59167	rpsL	30S ribosomal protein S12	Translation	x						x	
Q8DS35	rpsM	30S ribosomal protein S13	Translation	x	x	x	x	x	x	x	x
Q8DWB3	rpsO	30S ribosomal protein S15	Translation	x						x	
Q8DUN9	rpsP	30S ribosomal protein S16	Translation	x	x	x	x	x	x	x	x
Q8DS22	rpsQ	30S ribosomal protein S17	Translation	x		x			x	x	
P66474	rpsR	30S ribosomal protein S18	Translation				x			x	
Q8DS18	rpsS	30S ribosomal protein S19	Translation	x	x		x	x	x	x	x
Q8DU30	rpsT	30S ribosomal protein S20	Translation			x					
P66531	rpsU	30S ribosomal protein S21	Translation							x	
Q8DTW9	rs1	Putative ribosomal protein S1 sequence specific DNA-binding protein	Translation	x	x	x	x	x	x	x	x
Q8DTF9	SMU_1388	Putative RNA helicase	Translation					x			
Q8DUN8	SMU_866	UPF0109 protein	Translation			x					x
Q8DUE2	SMU_993	Ribosome biogenesis GTPase A	Translation	x							
Q8DS12	tsf	Elongation factor Ts	Translation	x	x	x	x	x	x	x	x
P72483	tuf	Elongation factor Tu	Translation	x	x	x	x	x	x	x	x
Transport											
Q8DT63	glnQ	Putative amino acid ABC transporter, ATP-binding protein	Amino acid transport	x	x	x	x	x		x	x
Q8DSU4	livF	Putative branched chain amino acid ABC transporter, ATP-binding protein	Amino acid transport							x	
Q8DSU3	livG	Putative branched chain amino acid ABC transporter, ATP-binding protein	Amino acid transport	x		x				x	
Q93DA2	metN	Methionine import ATP-binding protein MetN	Amino acid transport	x							
Q8DU84	opuAa	Putative ABC transporter, ATP-binding protein, proline/glycine betaine transport system	Amino acid transport	x			x			x	
Q8DRU8	opuCa	Putative osmoprotectant amino acid ABC transporter, ATP-binding protein	Amino acid transport				x				x
I6L912	SMU_1942c	Putative amino acid binding protein	Amino acid transport	x	x	x	x	x	x	x	
Q8DW36	SMU_241c	Putative ABC transporter, ATP-binding protein amino acid transport system	Amino acid transport	x							
Q8DUT8	SMU_805c	Putative amino acid ABC transporter, ATP-binding protein	Amino acid transport			x			x		
Q8DUT1	SMU_815	Putative amino acid transporter, amino acid-binding protein	Amino acid transport		x	x	x			x	
Q8DUS9	SMU_817	Putative amino acid transporter, amino acid-binding protein	Amino acid transport		x	x	x		x		
Q8DSZ8	lguL	Putative lactoylglutathione lyase	Metal ion binding	x	x	x	x	x	x	x	x

Q8DSF0	secA	Protein translocase subunit SecA	Protein transport	x	x					x	
Q8DUN3	fruI	Inducible fructose permease	Sugar transport								
Q8DT28	malX	Putative maltose/maltodextrin ABC transporter, sugar-binding protein MalX	Sugar transport	x	x	x	x	x	x		x
Q00749	msmE	Multiple sugar-binding protein	Sugar transport		x						
Q00752	msmK	Multiple sugar-binding transport ATP-binding protein MsmK	Sugar transport					x			
Q02420	mtlF	Mannitol-specific phosphotransferase enzyme IIA componen	Sugar transport	x							
Q8DT01	ptcB	Putative PTS system, cellobiose-specific IIB component	Sugar transport			x	x				
Q8DSC4	ptnA	Putative PTS system, mannose-specific component IIAB	Sugar transport	x	x		x	x	x	x	x
Q8DS05	ptsG	Putative PTS system, glucose-specific IIABC component	Sugar transport	x	x	x	x	x	x	x	x
P45595	ptsI	Phosphoenolpyruvate-protein phosphotransferase	Sugar transport	x	x	x	x	x	x	x	x
I6L910	ptxB	Putative PTS system, enzyme IIB component	Sugar transport		x						
P12655	scrA	PTS system sucrose-specific EIIBCA component	Sugar transport	x	x				x	x	
Q8DSC2	SMU_1879	Putative PTS system, mannose-specific component IID	Sugar transport							x	
Q8DS75	SMU_1958c	Putative PTS system, mannose-specific IIC component	Sugar transport	x				x		x	
Q8DS74	SMU_1960c	Putative PTS system, mannose-specific IIB component	Sugar transport	x	x	x	x	x	x	x	x
Q8DS73	SMU_1961c	Putative PTS system, sugar-specific enzyme IIA component	Sugar transport	x	x	x	x	x	x	x	x
P95788	atpG	ATP synthase gamma chain	Transport							x	
Q8DS49	comYA	Putative ABC transporter, ATP-binding protein ComYA late competence protein	Transport	x							
Q8DUA8	glrA	Putative ABC transporter, ATP-binding protein	Transport							x	
I6L926	lemA	Putative cytoplasmic membrane protein LemA-like protein	Transport		x				x		
Q8DSU0	livK	Putative ABC transporter, branched chain amino acid-binding protein	Transport	x	x	x	x	x	x	x	x
Q8DW25	oppA	Putative oligopeptide ABC transporter, substrate-binding protein OppA	Transport	x	x	x	x	x	x	x	x
Q8DU54	opuBa	Putative ABC transporter, ATP-binding protein, choline transporter	Transport	x						x	
I6L8X8	rgpD	Putative polysaccharide ABC transporter, ATP-binding protein	Transport							x	
Q8DSH4	scnF	Putative bacteriocin component ScnF-like protein, putative ABC transporter, ATP-binding protein	Transport			x	x				
Q8DW86	sloA	Putative ABC transporter, ATP-binding protein possible iron and/or manganese ABC transport system	Transport	x	x		x	x	x	x	x
Q8DUA2	SMU_1041	Putative ABC transporter, ATP-binding protein	Transport	x						x	
Q8DU71	SMU_1078c	Putative ABC transporter, ATP-binding protein	Transport		x						
Q8DU57	SMU_1093	Putative ABC transporter, permease protein	Transport		x						x

Q8DU36	SMU_1121c	Putative ABC transporter	Transport	x	x	x	x	x	x	x	x
Q8DTZ7	SMU_1163c	Putative ABC transporter, ATP-binding protein	Transport					x			
Q8DTZ4	SMU_1166c	Putative ABC transporter, permease protein	Transport					x	x		
Q8CM14	SMU_1348c	Putative ABC transporter ATP-binding protein	Transport		x	x	x		x	x	x
Q8DTD8	SMU_1412c	Putative ABC transporter, membrane protein subunit and ATP-binding protein	Transport						x		
Q8DT62	SMU_1520	Putative ABC transporter, glutamine binding protein	Transport	x	x	x	x	x	x	x	x
Q8DSZ6	SMU_1605	Putative MDR permease possible transmembrane efflux protein	Transport		x						
I6L8Y2	SMU_1927	Putative ABC transporter, ATP-binding protein	Transport			x					
Q8DRZ6	SMU_2057c	Putative cadmium-transporting ATPase P-type ATPase	Transport					x			
Q8DRY9	SMU_2066c	Putative transmembrane protein	Transport				x				
Q8DW32	SMU_247	Putative ABC transporter ATP-binding protein	Transport	x	x	x	x	x	x	x	x
Q8DW31	SMU_248	Putative ABC transporter, membrane protein	Transport	x	x			x		x	x
Q8DW05	SMU_286	Putative ABC transporter ATP-binding protein ComA	Transport				x	x			
Q8DVM1	SMU_459	Putative ABC transporter, amino acid binding protein	Transport						x	x	
Q8DUT7	SMU_806c	Putative glutamine ABC transporter, permease protein	Transport	x	x	x	x	x	x	x	x
Q8DUP0	SMU_864	Putative ABC transporter, permease component	Transport			x					x
Q8DUL7	SMU_902	Putative ABC transporter, ATP-binding protein	Transport	x							
Q8DUK5	SMU_922	Putative ABC transporter, ATP-binding protein	Transport					x			
Q8DUD7	SMU_998	Putative ABC transporter, periplasmic ferrichrome-binding protein	Transport	x	x	x	x	x	x	x	x
Q8DSR3	trkA	Putative potassium uptake system protein TrkA	Transport					x			
Q8DT34	trkB	Putative potassium uptake system protein TrkB	Transport					x			
Uncharacterized/Unknown											
Q8DVN1	MU_448	Uncharacterized protein	Uncharacterized	x				x			
Q8DVH8	MU_510c	Uncharacterized protein	Uncharacterized	x	x	x	x	x	x		x
Q8DUC6	SMU_1014	Uncharacterized protein	Uncharacterized							x	
Q8DU92	SMU_1052	Uncharacterized protein	Uncharacterized								
Q8DU59	SMU_1090	Uncharacterized protein	Uncharacterized	x	x		x		x		x
Q8DU18	SMU_1140c	Uncharacterized protein	Uncharacterized						x		

Q8DTW8	SMU_1201c	Uncharacterized protein	Uncharacterized	x	x	x	x	x	x	x	x
Q8DTW2	SMU_1208c	Uncharacterized protein	Uncharacterized		x						
Q8DWD8	SMU_125	Uncharacterized protein	Uncharacterized				x				x
Q8DTL2	SMU_1323	Uncharacterized protein	Uncharacterized			x	x	x	x		x
Q8DTG7	SMU_1377c	Uncharacterized protein	Uncharacterized	x	x		x	x	x	x	x
Q8DTG6	SMU_1378	Uncharacterized protein	Uncharacterized		x						
Q8DTB5	SMU_1442c	Uncharacterized protein	Uncharacterized	x	x	x	x	x	x	x	x
Q8DTB0	SMU_1447c	Uncharacterized protein	Uncharacterized				x			x	
Q8DT86	SMU_1479	Uncharacterized protein	Uncharacterized	x			x				
Q8DSW7	SMU_1641c	Uncharacterized protein	Uncharacterized	x	x	x	x	x	x	x	x
Q8DWA2	SMU_165	Uncharacterized protein	Uncharacterized						x	x	
Q8DSS8	SMU_1681c	Uncharacterized protein	Uncharacterized	x			x	x		x	x
Q8DSM0	SMU_1760c	Uncharacterized protein	Uncharacterized	x	x	x	x	x	x	x	x
Q8DSL9	SMU_1761c	Uncharacterized protein	Uncharacterized				x			x	
Q8DSL6	SMU_1764c	Uncharacterized protein	Uncharacterized							x	
Q8DSE8	SMU_1846c	Uncharacterized protein	Uncharacterized	x	x	x	x	x	x	x	x
Q8DSC9	SMU_1872c	Uncharacterized protein	Uncharacterized	x			x				
Q8DSA4	SMU_1904c	Uncharacterized protein	Uncharacterized	x	x	x	x	x	x	x	x
I6L8Z9	SMU_1925c	Uncharacterized protein	Uncharacterized			x					
Q8DS83	SMU_1946	Uncharacterized protein	Uncharacterized			x					
Q8DW70	SMU_205c	Uncharacterized protein	Uncharacterized	x			x		x		
Q8DRZ2	SMU_2061	Uncharacterized protein	Uncharacterized			x			x		x
Q8DRX7	SMU_2079c	UPF0297 protein	Uncharacterized	x	x	x	x	x	x	x	x
Q8DW66	SMU_209c	Uncharacterized protein	Uncharacterized	x	x	x	x	x	x	x	x
Q8DW65	SMU_210c	Uncharacterized protein	Uncharacterized	x	x	x	x	x	x	x	x
Q8DRS2	SMU_2147c	Uncharacterized protein	Uncharacterized			x				x	
Q8DW41	SMU_235	Uncharacterized protein	Uncharacterized	x	x	x	x	x	x	x	x
Q8DVX7	SMU_332	Uncharacterized protein	Uncharacterized				x				
Q8DVX6	SMU_333	Uncharacterized protein	Uncharacterized			x		x			x

Q8DVU4	SMU_371	Uncharacterized protein	Uncharacterized								x
Q8DVS5	SMU_392c	Uncharacterized protein	Uncharacterized				x				
Q8DVS4	SMU_393	Uncharacterized protein	Uncharacterized		x	x	x	x			x
Q8DVQ5	SMU_415	Uncharacterized protein	Uncharacterized	x			x				
Q8DVP5	SMU_428	Uncharacterized protein	Uncharacterized						x		
Q8DVI6	SMU_502	Uncharacterized protein	Uncharacterized				x		x		
Q8DVG3	SMU_527	Uncharacterized protein	Uncharacterized		x						
Q8DVC8	SMU_564	Uncharacterized protein	Uncharacterized	x	x	x	x	x	x	x	x
Q8DVA9	SMU_586	Uncharacterized protein	Uncharacterized		x		x				
Q8DVA5	SMU_591c	Uncharacterized protein	Uncharacterized	x							
Q8DV71	SMU_635	Uncharacterized protein	Uncharacterized								x
Q8DWI5	SMU_63c	Uncharacterized protein	Uncharacterized				x		x	x	
Q8DV31	SMU_685	Uncharacterized protein	Uncharacterized	x					x	x	
Q8DV27	SMU_690	Uncharacterized protein	Uncharacterized		x						
Q8DUY0	SMU_751	Uncharacterized protein	Uncharacterized	x						x	x
Q8DUX7	SMU_757	Uncharacterized protein	Uncharacterized		x		x		x		
Q8DUX2	SMU_768	Uncharacterized protein	Uncharacterized	x		x	x		x	x	x
Q8DUP2	SMU_862	Uncharacterized protein	Uncharacterized								
Q8DUM1	SMU_898	Uncharacterized protein	Uncharacterized	x			x		x	x	x
Q8DTJ0	SMU_1347c	Uncharacterized protein	Unknown	x				x		x	
Q8DRT3	SMU_2133c	Putative membrane protein	Unknown			x					
Q8DVU6	SMU_369c	UPF0356 protein	Unknown	x	x	x	x	x		x	x
Q8DVN2	SMU_447	UPF0291 protein	Unknown	x	x	x	x	x	x	x	x
Q8DWH8	SMU_72	UPF0237 protein	Unknown	x	x	x	x	x	x	x	x
Q8DUV9	SMU_782	UPF0342 protein	Unknown	x	x	x	x	x	x		x
