# Supplementary material for: Engineered Salivary Peptides Reduce Enamel Demineralization Provoked by Cariogenic S. mutans Biofilm
Source: Microorganisms. 2022 Mar 30;10(4):742. doi: 10.3390/microorganisms10040742 (PMC9032980; doi:10.3390/microorganisms10040742)
Supplement: Supplementary file 1 [file microorganisms-10-00742-s001.zip › S3_Table.pdf]

Supplementary Table S3. List of cellular proteins identified when the proteins/peptides were used to form the AEP and as daily treatments (experimental condition 2).

Intracellular proteins

Accession	Gene name	Protein name	Protein function	Treatment							
				A	B	C	D	E	F	G	H
Adaptative responses to environmental changes											
Q8DTJ8	bacD	Putative bacitracin synthetase	Antibiotic biosynthesis				x	x			
Q8DWD4	adhD	Dihydrolipoyl dehydrogenase	Cell redox homeostasis			x		x			
Q8DTE3	cas9	CRISPR-associated endonuclease Cas9	CRISP element metabolism					x			
Q8DVY2	radA	DNA repair protein RadA	DNA repair	x							x
P27624	recA	Protein RecA	DNA repair			x	x				
Q8DSC6	recD2	ATP-dependent RecD-like DNA helicase	DNA repair		x						
Q8DT75	rexB	ATP-dependent helicase/deoxyribonuclease subunit B	DNA repair						x		
Q9AIV4	smnA	Nuclease SmnA	DNA repair						x		
P72481	uvrA	UvrABC system protein A	DNA repair								x
Q8DSP2	SMU_1733c	Putative SNF helicase	DNA replication, recombination and repair								
Q8DW67	SMU_208c	Putative transposon protein possible DNA segregation ATPase	Recombination	x							
Q8DU29	ciaH	Putative histidine kinase sensor CiaH	Signal transduction						x		
Q8DSH1	scnK	Putative histidine kinase, ScnK-like protein	Signal transduction								
Q8DV79	SMU_626	Putative competence protein	Transformation							x	
Amino acid metabolism and biosynthesis											
Q8DUW2	aroB	3-dehydroquinate synthase	Amino acid biosynthesis								x
P10539	asd	aspartate-semialdehyde dehydrogenase	Amino acid biosynthesis			x			x		
Q8DUP3	carB	Carbamoyl-phosphate synthase large chain	Amino acid biosynthesis						x		
Q8DW43	ilvC	Ketol-acid reductoisomerase (NADP(+))	Amino acid biosynthesis	x	x	x	x	x	x	x	x
Q8DTV5	nylA	Putative amidase	Amino acid biosynthesis						x		
Q8DSV3	serC	Phosphoserine aminotransferase	Amino acid biosynthesis			x					
Q8DUW0	SMU_781	Putative prephenate dehydrogenase	Amino acid biosynthesis				x				

Q8DUH7	SMU_952	Putative methyltransferase	Amino acid biosynthesis						x		
Bacterial adherence and biofilm formation											
Q8DU58	wapE	Uncharacterized protein	Adherence			x					
Q8DVR0	brpA	Biofilm regulatory protein A	Biofilm formation		x	x					x
Q8DTF1	gbpC	Glucan-binding protein C, GbpC	Biofilm formation			x				x	
Q8DUW9	gbpD	Glucan-binding protein D with lipase activity BglB-like protein	Biofilm formation			x					
P23504	spaP	Cell surface antigen I/II	Cell wall antigen	x	x	x	x	x	x	x	x
P11701	ftf	Levansucrase	EPS biosynthesis		x						
P08987	gtfB	Glucosyltransferase-I	EPS biosynthesis	x	x	x	x		x		x
P13470	gtfC	Glucosyltransferase-SI	EPS biosynthesis	x	x	x	x		x		
P49331	gtfD	Glucosyltransferase-S	EPS biosynthesis			x		x	x		x
Q8DUS4	rgpA	Putative RgpAc glycosyltransferase	EPS biosynthesis							x	
Carbohydrate metabolism and energy production											
Q8DT55	phsG	Alpha-1,4 glucan phosphorylase	Carbohydrate metabolic process			x					
Q03174	fruA	Fructan beta-fructosidase	Carbohydrate metabolic process								x
P96994	galT	Galactose-1-phosphate uridylyltransferase	Carbohydrate metabolic process						x		
Q8DVV3	gapC	Glyceraldehyde-3-phosphate dehydrogenase	Carbohydrate metabolic process	x	x	x	x	x	x	x	x
Q8DT31	glgP	Alpha-1,4 glucan phosphorylase	Carbohydrate metabolic process	x	x				x		x
Q8DTC6	glmM	Phosphoglucosamine mutase	Carbohydrate metabolic process								x
P26424	lacB	Galactose-6-phosphate isomerase subunit LacB	Carbohydrate metabolic process	x					x		
Q59934	pfl	Formate acetyltransferase	Carbohydrate metabolic process			x	x		x		
Q8CWY1	pfl2	Formate acetyltransferase (Pyruvate formate-lyase 2)	Carbohydrate metabolic process	x		x	x	x			
Q8DU72	pgm	Putative phosphoglucomutase	Carbohydrate metabolic process		x						
P95780	rmlB	dTDP-glucose 4,6-dehydratase	Carbohydrate metabolic process			x		x			
Q8DWF5	SMU_104	Putative alpha-glucosidase glycosyl hydrolase	Carbohydrate metabolic process			x					
O68579	ppaC	Probable manganese-dependent inorganic pyrophosphatase	Energy metabolism	x	x	x	x	x	x		x
Q8DTS9	eno	Enolase	Glycolytic process	x	x	x	x	x	x	x	x

Q8DWG0	fbaA	Fructose-1,6-biphosphate aldolase	Glycolytic process	x	x	x	x	x	x	x	x
P26283	ldh	L-lactate dehydrogenase	Glycolytic process	x	x		x	x	x		x
Q8DVV2	pgk	Phosphoglycerate kinase	Glycolytic process	x	x	x	x	x	x	x	x
Q8DTX7	pykF	Pyruvate kinase	Glycolytic process	x	x	x	x	x	x	x	x
Cell division, replication and cell wall synthesis											
Q8DVD4	divIVA	Putative cell division protein DivIVA	Cell division			x		x			
Q8DSP6	mltG	Endolytic murein transglycosylase	Cell wall biosynthesis						x		
Q53526	dltA	D-alanine--D-alanyl carrier protein ligase	Cell wall biosynthesis		x						
Q8DSS4	dltD	Protein DltD	Cell wall biosynthesis		x						
Q8DVM5	pbp2x	Putative penicillin-binding protein 2X	Cell wall biosynthesis	x	x	x	x				
Q8DS45	pbp1b	Putative membrane carboxypeptidase, penicillin-binding protein 1b	Cell wall biosynthesis	x	x			x			
Q8DVA0	pbp2b	Penicillin-binding protein 2b	Cell wall biosynthesis			x					
Q8DVE2	murG	UDP-N-acetylglucosamine--N-acetylmuramyl-pyrophosphoryl-undecaprenol N-acetylglucosamine transferase	Peptidoglycan biosynthesis			x					
Q8DUJ1	SMU_937	Putative mevalonate diphosphate decarboxylase	Peptidoglycan biosynthesis								x
Q8DTX5	dnaE	DNA-directed DNA polymerase	Replication	x							
Q8DT49	ligA	DNA ligase	Replication					x	x	x	x
Q8DTY6	pcrA	ATP-dependent DNA helicase	Replication			x					
Nucleoside/Nucleotide metabolism and biosynthesis											
P95785	atpF	ATP synthase subunit b	Nucleotide biosynthesis	x							
Q8DWM2	prs1	Ribose-phosphate pyrophosphokinase 1	Nucleotide biosynthesis	x			x				
Q8DWL5	purL	Phosphoribosylformylglycinamidine synthase	Nucleotide biosynthesis								x
I6L8Y1	SMU_273	Putative hexulose-6-phosphate synthase	Nucleotide biosynthesis								x
Q8DVL6	SMU_464	Nicotinate phosphoribosyltransferase	Nucleotide biosynthesis		x						x
Q8DU63	tdk	Thymidine kinase	Nucleotide biosynthesis								x
Q8DST6	upp	Uracil phosphoribosyltransferase	Nucleotide biosynthesis				x				
Other metabolic processes											

Q8DWB9	adhE	Aldehyde-alcohol dehydrogenase	Alcohol metabolic process	x					x		x
Q8DTJ6	SMU_1341c	Putative gramicidin S synthetase	Catalytic activity				x				
Q9XB21	hup	DNA-binding protein HU	Chromosome condensation	x	x	x	x	x	x	x	x
Q7ZAL0	smc	Chromosome partition protein Smc	Chromosome condensation						x		x
Q8DSN3	acp	acyl carrier protein	Fatty acid biosynthesis		x				x		
Q8DUC1	cilA	Citrate lyase alpha chain	Fatty acid biosynthesis					x			
Q8DWD5	adhC	Dihydrolipoamide acetyltransferase component of pyruvate dehydrogenase complex	Metabolic processes				x				
Q8DTJ5	bacA1	Putative bacitracin synthetase 1 BacA	Metabolic processes				x			x	x
Q8CWW8	fabD	Malonyl CoA-acyl carrier protein transacylase	Metabolic processes						x		
Q8DT10	SMU_1588c	Putative hexosyltransferase	Metabolic processes			x					
Q8DSH9	SMU_1806	Putative glycosyltransferase	Metabolic processes	x							
Q8DRZ7	SMU_2056	Putative ATPase	Metabolic processes						x		
Q8DW46	SMU_229	Uncharacterized protein	Metabolic processes					x	x		
Q8DVN8	SMU_438c	Putative (R)-2-hydroxyglutaryl-CoA dehydratase activator-related protein	Metabolic processes				x				
Q8DVF0	dpr	Peroxide resistance protein Dpr	Oxidation-reduction process	x	x	x	x	x	x	x	x
Q59931	gapN	NADP-dependent glyceraldehyde-3-phosphate dehydrogenase	Oxidation-reduction process	x	x	x	x	x	x	x	x
Q8CWY9	gltA	Glutamate synthase (Large subunit)	Oxidation-reduction process			x				x	
Q8CZF1	pdhB	Putative pyruvate dehydrogenase E1 component beta subunit	Oxidation-reduction process			x				x	
Q8DW88	urdA	Urocanate reductase	Oxidation-reduction process		x		x				
Q8DST7	clpP	ATP-dependent Clp protease proteolytic subunit	Proteolysis					x			
Q8DWM7	ftsH	ATP-dependent zinc metalloprotease FtsH	Proteolysis				x				x
Q8DRQ6	htrA	Serine protease HtrA	Proteolysis		x						
Q8DS80	pbp2a	Putative membrane carboxypeptidase, penicillin-binding protein 2a	Proteolysis			x					
Q8DVS2	pepX	Xaa-Pro dipeptidyl-peptidase	Proteolysis				x		x		
Q8DRR6	SMU_2153c	Putative peptidase	Proteolysis						x		
Q8DWB2	pnp	Polyribonucleotide nucleotidyltransferase	RNA degradation								x
Q8DUB2	SMU_1030	Putative polyribonucleotide nucleotidyltransferase Tn916 ORF8-like	RNA degradation	x							
Q59938	acn	Aconitate hydratase A	Tricarboxylic acid cycle	x							
Q8DV10	ppc	Phosphoenolpyruvate carboxylase	Tricarboxylic acid cycle			x					

Transcription

Q8DUH3	clp	Putative Clp-like ATP-dependent protease, ATP-binding subunit	Transcription	x	x	x	x	x	x	x	x
Q8DVD0	clpE	ATP-dependent protease ClpE	Transcription			x					
Q8DSP7	greA	Transcription elongation factor GreA	Transcription	x			x				
Q02425	mtlR	Putative transcriptional regulator MtlR	Transcription		x				x		
Q8DSE9	nusB	Transcription antitermination protein NusB	Transcription								x
Q8DS36	rpoA	DNA-directed RNA polymerase subunit alpha	Transcription			x		x			
Q8DS47	rpoC	DNA-directed RNA polymerase subunit beta'	Transcription	x	x						
O33662	sigA	RNA polymerase sigma factor SigA	Transcription					x			
Q8DUB7	SMU_1025	Putative transcriptional regulator	Transcription	x							
Q8DWE9	SMU_112c	Putative transcriptional regulator	Transcription			x					
Q8DWD9	SMU_124	Putative transcriptional regulator (MarR family)	Transcription		x	x					
Q8DTE1	SMU_1409c	Putative transcriptional regulator	Transcription							x	
Q8DSJ4	SMU_1789c	Probable transcriptional regulatory protein SMU_1789c	Transcription			x					
Q8DSH3	SMU_1812	Putative transposase, ISSmu2	Transcription								x
Q8DS71	SMU_1964c	Putative response regulator	Transcription	x							
I6L8Y6	SMU_309	Regulator of sorbitol operon	Transcription	x							
Q8DVJ5	SMU_491	Putative DeoR-type transcriptional regulator	Transcription					x			

Translation and protein synthesis

Q8CWY0	alaS	Alanine--tRNA ligase	Protein biosynthesis	x							
Q8DRW2	argS	Arginine--tRNA ligase	Protein biosynthesis								x
Q8DRV9	aspS2	Aspartate--tRNA ligase 2	Protein biosynthesis								x
Q8CWY5	glyS	Glycine--tRNA ligase beta subunit	Protein biosynthesis		x						
Q8DVD3	ileS	Isoleucine--tRNA ligase	Protein biosynthesis		x						
Q8CWX2	pheT	Phenylalanine--tRNA ligase beta subunit	Protein biosynthesis	x							
Q8DWN6	ychF	Ribosome-binding ATPase YchF	Protein biosynthesis		x						
Q8DS14	clpC	Class III stress response-related ATP-dependent Clp protease, ATP-binding subunit	Protein folding		x						

Q8DWH2	dnaJ	Chaperone protein DnaJ	Protein folding				x				
O06942	dnaK	Chaperone protein DnaK	Protein folding	x	x	x	x	x	x	x	x
Q8CWW6	groL	60 kDa chaperonin	Protein folding	x	x	x	x	x	x	x	x
Q8CWW5	groS	10 kDa chaperonin	Protein folding		x	x			x		x
Q8CVC6	prsA	Foldase protein PrsA	Protein folding	x		x		x	x		x
Q8CWZ6	tig	Trigger factor	Protein folding	x	x	x	x	x	x	x	x
Q54431	ffh	Signal recognition particle protein	Protein targeting								x
Q8CWX8	ftsY	Signal recognition particle receptor FtsY	Protein targeting	x							
Q8DVV4	fusA	Elongation factor G	Translation	x	x	x	x	x	x	x	x
Q8DSG5	gatA	Glutamyl-tRNA(Gln) amidotransferase subunit A	Translation		x			x			
Q8DSG6	gatB	Aspartyl/glutamyl-tRNA(Asn/Gln) amidotransferase subunit B	Translation	x	x	x	x	x	x	x	x
Q8DVI8	hpf	Ribosome hibernation promoting factor	Translation		x	x	x	x	x		
Q8DVP9	infB	Translation initiation factor IF-2	Translation								x
Q8DTF3	lepA	Elongation factor 4	Translation	x							
Q8DTT8	mnmE	tRNA modification GTPase MnmE	Translation								x
Q8DSY0	rplA	50S ribosomal protein L1	Translation		x						
Q8DS17	rplD	50S ribosomal protein L4	Translation					x			x
Q8DUH2	rplJ	50S ribosomal protein L10	Translation						x		
Q8DUG9	rplL	50S ribosomal protein L7/L12	Translation	x	x	x	x	x	x	x	x
Q8DS20	rplP	50S ribosomal protein L16	Translation			x	x				
Q8DS37	rplQ	50S ribosomal protein L17	Translation	x		x		x			
Q8DS29	rplR	50S ribosomal protein L18	Translation						x		
Q8DUQ6	rplU	50S ribosomal protein L21	Translation	x		x					
Q8DS24	rplX	50S ribosomal protein L24	Translation	x	x	x	x	x	x	x	x
Q8DS11	rpsB	30S ribosomal protein S2	Translation	x	x	x	x	x	x	x	x
P59186	rpsC	30S ribosomal protein S3	Translation			x	x	x	x		
P59133	rpsD	30S ribosomal protein S4	Translation		x			x	x	x	x
Q8DSD7	rpsF	30S ribosomal protein S6	Translation		x			x			
Q8DVV5	rpsG	30S ribosomal protein S7	Translation								

Q8DW97	rpsI	30S ribosomal protein S9	Translation	x		x	x	x			
Q8DS18	rpsS	30S ribosomal protein S19	Translation					x			
Q8DTW9	rs1	Putative ribosomal protein S1 sequence specific DNA-binding protein	Translation	x	x	x	x	x	x		x
Q8DS12	tsf	Elongation factor Ts	Translation	x	x	x	x		x		x
P72483	tuf	Elongation factor Tu	Translation	x	x	x	x	x	x	x	x
Q8CWW9	vacB	Ribonuclease R	Translation	x							
Transport											
Q8DRU8	opuCa	Putative osmoprotectant amino acid ABC transporter, ATP-binding protein	Amino acid transport			x					
Q8DVC5	SMU_568	Putative amino acid ABC transporter, ATP-binding protein	Amino acid transport				x				
Q8DUT1	SMU_815	Putative amino acid transporter, amino acid-binding protein	Amino acid transport	x							
Q8DSZ8	lguL	Putative lactoylglutathione lyase	Metal ion binding			x					
Q8DU24	pstB1	Phosphate import ATP-binding protein PstB 1	Phosphate transport				x				
Q8DSF0	secA	Protein translocase subunit SecA	Protein transport				x				
Q8DUN3	fruI	Inducible fructose permease	Sugar transport	x				x		x	x
Q8DT27	malF	Putative maltose/maltodextrin ABC transporter, permease protein MalF	Sugar transport								
Q8DT28	malX	Putative maltose/maltodextrin ABC transporter, sugar-binding protein MalX	Sugar transport		x	x	x	x	x		
Q8DSC4	ptnA	Putative PTS system, mannose-specific component IIAB	Sugar transport			x		x			
P45595	ptsI	Phosphoenolpyruvate-protein phosphotransferase	Sugar transport	x							
Q8DS74	SMU_1960c	Putative PTS system, mannose-specific IIB component	Sugar transport	x	x	x	x	x	x	x	x
Q8DS69	SMU_1966c	Putative periplasmic sugar-binding protein	Sugar transport	x							
P95788	atpG	ATP synthase gamma chain	Transport						x		
Q8DSU0	livK	Putative ABC transporter, branched chain amino acid-binding protein	Transport			x					
Q8DU85	opuAb	Putative ABC transporter, proline/glycine betaine permease protein	Transport		x						
Q8DRU6	opuCc	Putative ABC transporter osmoprotectant-binding protein, glycine betaine/carnitine/choline ABC transporter	Transport								x
Q8DT32	pacL	Putative cation-transporting P-type ATPase PacL	Transport			x					
Q8DS87	psaB	Putative ABC transporter, permease protein	Transport		x						
Q8DSH4	scnF	Putative bacteriocin component ScnF-like protein, putative ABC transporter, ATP-binding protein	Transport				x				
Q8DSH6	scnG	Putative bacteroiocin operon protein ScnG-like protein	Transport	x							

Q8DU70	SMU_1079c	Putative ABC transporter, ATP-binding protein	Transport								
Q8DU57	SMU_1093	Putative ABC transporter, permease protein	Transport	x			x				x
Q8DTZ7	SMU_1163c	Putative ABC transporter, ATP-binding protein	Transport						x		
Q8DTZ4	SMU_1166c	Putative ABC transporter, permease protein	Transport	x							
Q8DTD8	SMU_1412c	Putative ABC transporter, membrane protein subunit and ATP-binding protein	Transport						x		x
Q8DTC1	SMU_1431c	Putative ABC transporter, ATP-binding protein	Transport	x							
Q8DSJ6	SMU_1787c	Putative secreted protein	Transport						x		
Q8DSA9	SMU_1898	Putative ABC transporter, ATP-binding and permease protein	Transport	x							
Q8DRZ6	SMU_2057c	Putative cadmium-transporting ATPase P-type ATPase	Transport	x							
I6L919	SMU_2109	Putative MDR permease possible multidrug efflux pump	Transport			x					
Q8DVR2	SMU_408	Putative permease	Transport		x				x		
Q8DVM1	SMU_459	Putative ABC transporter, amino acid binding protein	Transport			x					
Q8DVG6	SMU_524	Putative ABC transporter, ATP-binding protein	Transport						x		
Q8DV57	SMU_651c	Putative ABC transporter, substrate-binding protein	Transport		x				x		
Q8DUT7	SMU_806c	Putative glutamine ABC transporter, permease protein	Transport						x		
Q8DUL7	SMU_902	Putative ABC transporter, ATP-binding protein	Transport								x
Q8DUK5	SMU_922	Putative ABC transporter, ATP-binding protein	Transport			x					
Q8DUK4	SMU_923	Putative ABC transporter, ATP-binding protein	Transport								x
Q8DUD7	SMU_998	Putative ABC transporter, periplasmic ferrichrome-binding protein	Transport							x	
Uncharacterized/Unknown											
Q8DU93	SMU_1051	Putative iron-sulfur cofactor synthesis protein NifS family	Uncharacterized				x				
Q8CWZ5	SMU_109	Uncharacterized protein	Uncharacterized		x						
Q8DU49	SMU_1104c	Uncharacterized protein	Uncharacterized								
Q8DU40	SMU_1116c	Uncharacterized protein	Uncharacterized	x							
Q8DU32	SMU_1125c	Uncharacterized protein	Uncharacterized				x				
Q8DTE2	SMU_1406c	Uncharacterized protein	Uncharacterized						x		
Q8DTC5	SMU_1427c	Uncharacterized protein	Uncharacterized								x
Q8DTB0	SMU_1447c	Uncharacterized protein	Uncharacterized					x	x		

Q8DT97	SMU_1465c	Uncharacterized protein	Uncharacterized	x							
Q8DT86	SMU_1479	Uncharacterized protein	Uncharacterized	x	x	x	x	x	x	x	x
Q8DT20	SMU_1576c	Uncharacterized protein	Uncharacterized								
Q8DT19	SMU_1577c	Uncharacterized protein	Uncharacterized				x			x	
Q8DWA2	SMU_165	Uncharacterized protein	Uncharacterized						x		
Q8DSP3	SMU_1732c	Uncharacterized protein	Uncharacterized								x
Q8DSM0	SMU_1760c	Uncharacterized protein	Uncharacterized	x	x	x	x			x	
Q8DW90	SMU_178	Uncharacterized protein	Uncharacterized				x				
Q8DSF8	SMU_1830c	Uncharacterized protein	Uncharacterized					x			
Q8DS78	SMU_1951c	Uncharacterized protein	Uncharacterized								x
Q8DW77	SMU_197c	Uncharacterized protein	Uncharacterized		x						
Q8DW70	SMU_205c	Uncharacterized protein	Uncharacterized								x
Q8DRX7	SMU_2079c	UPF0297 protein	Uncharacterized							x	
Q8DRV8	SMU_2104	Uncharacterized protein	Uncharacterized					x			
Q8DW41	SMU_235	Uncharacterized protein	Uncharacterized	x	x	x	x	x	x	x	x
Q8DW07	SMU_284	Uncharacterized protein	Uncharacterized						x		
Q8DVV8	SMU_354	Uncharacterized protein	Uncharacterized								x
Q8DWJ6	SMU_49	Uncharacterized protein	Uncharacterized	x							
Q8DVA9	SMU_586	Uncharacterized protein	Uncharacterized	x							x
Q8DVA7	SMU_588	Uncharacterized protein	Uncharacterized	x							
Q8DV71	SMU_635	Uncharacterized protein	Uncharacterized						x		
Q8DWI5	SMU_63c	Uncharacterized protein	Uncharacterized	x	x	x	x		x		
Q8DV34	SMU_682	Uncharacterized protein	Uncharacterized	x						x	
Q8DUY9	SMU_739c	Uncharacterized protein	Uncharacterized	x			x	x		x	
Q8DUU7	SMU_796	Uncharacterized protein	Uncharacterized		x						
Q8DUP2	SMU_862	Uncharacterized protein	Uncharacterized								x
Q8DTJ0	SMU_1347c	Uncharacterized protein	Unknown	x			x	x			
Q8DST3	SMU_1676c	Putative membrane protein	Unknown			x					
Q8DRT3	SMU_2133c	Putative membrane protein	Unknown	x			x		x	x	x

Q8DUV9	SMU_782	UPF0342 protein	Unknown	x	x	x		x	x	x
