# Supplementary material for: Engineered Salivary Peptides Reduce Enamel Demineralization Provoked by Cariogenic S. mutans Biofilm
Source: Microorganisms. 2022 Mar 30;10(4):742. doi: 10.3390/microorganisms10040742 (PMC9032980; doi:10.3390/microorganisms10040742)
Supplement: Supplementary file 1 [file microorganisms-10-00742-s001.zip › S4_Table.pdf]

Supplementary Table S4. List of ECM proteins identified when the proteins/peptides were used to form the AEP and as daily treatments (experimental condition 2).

ECM proteins											
**Accession**	**Gene name**	**Protein name**	**Protein function**	**Treatment**							
				**A**	**B**	**C**	**D**	**E**	**F**	**G**	**H**
Adaptative responses to environmental changes											
I6L8Y0	ahpC	alkyl hydroperoxide reductase	Cell redox homeostasis				x				
Q8DUK3	tpx	Thiol peroxidase	Cell redox homeostasis	x			x		x	x	x
Q8DSD2	trxA	Thioredoxin	Cell redox homeostasis	x	x	x	x	x	x	x	
P09738	sodA	Superoxide dismutase [Mn/Fe]	Cellular detoxification	x	x	x	x	x	x	x	x
Q8DTE3	cas9	CRISPR-associated endonuclease Cas9	CRISP element metabolism	x	x						
Q8DRW8	mutS	DNA mismatch repair protein MutS	DNA repair		x						
P27624	recA	Protein RecA	DNA repair	x	x	x	x	x	x	x	x
P72481	uvrA	UvrABC system protein A	DNA repair					x			
Q8DSD8	ssb	Single-stranded DNA-binding protein	DNA replication, recombination and repair	x	x	x	x	x	x		x
Q8DSU5	SMU_1664c	Putative acetoin utilization protein, acetoin dehydrogenase	Energy store	x			x	x	x		x
Q8DSJ5	SMU_1788c	Putative bacterocin transport accessory protein, Bta	Killing factor	x			x		x		
Q8DW76	SMU_198c	Putative conjugative transposon protein	Recombination		x	x					
Q8DU29	ciaH	Putative histidine kinase sensor CiaH	Signal transduction					x			
I6L903	SMU_1009	Putative histidine kinase	Signal transduction	x					x		
Q8DW95	SMU_173	Putative ppGpp-regulated growth inhibitor	Stress	x		x	x				
Q8DSF9	SMU_1828	Universal stress protein	Stress	x	x	x	x	x	x		x
Amino acid metabolism and biosynthesis											
Q8DUV8	aroA	3-phosphoshikimate 1-carboxyvinyltransferase	Amino acid biosynthesis		x	x	x	x			
Q8DUW4	aroD	3-dehydroquinate dehydratase	Amino acid biosynthesis				x				
Q8DUW3	aroE	Shikimate dehydrogenase (NADP(+))	Amino acid biosynthesis	x	x	x	x	x	x	x	x
P10539	asd	aspartate-semialdehyde dehydrogenase	Amino acid biosynthesis			x			x		
Q8DTM1	aspB	Asparagine--oxo-acid transaminase	Amino acid biosynthesis		x						x

Q8DUP3	carB	Carbamoyl-phosphate synthase large chain	Amino acid biosynthesis	x							
Q8DUL9	dapB	4-hydroxy-tetrahydrodipicolinate reductase	Amino acid biosynthesis				x				
Q8DVY7	dapH	2,3,4,5-tetrahydropyridine-2,6-dicarboxylate N-acetyltransferase	Amino acid biosynthesis	x			x	x			
Q8DVU9	glnA	Glutamine synthetase type 1 glutamate--ammonia ligase	Amino acid biosynthesis	x	x	x	x	x	x	x	x
Q8CWY8	gltB	NADPH-dependent glutamate synthase (Small subunit)	Amino acid biosynthesis		x						
Q8DU67	glyA	Serine hydroxymethyltransferase	Amino acid biosynthesis				x				
Q8DW42	ilvA	L-threonine dehydratase	Amino acid biosynthesis		x		x				
Q8DW45	ilvB	Acetolactate synthase	Amino acid biosynthesis								x
Q8DW43	ilvC	Ketol-acid reductoisomerase (NADP(+))	Amino acid biosynthesis	x	x	x	x	x	x	x	x
Q8DRT7	ilvD	Dihydroxy-acid dehydratase	Amino acid biosynthesis			x					
Q8DTW7	ilvE	Branched-chain-amino-acid aminotransferase	Amino acid biosynthesis	x	x	x	x	x	x	x	x
Q8DW44	ilvH	Acetolactate synthase, small subunit	Amino acid biosynthesis						x		
Q8DTG2	leuA	2-isopropylmalate synthase	Amino acid biosynthesis	x	x	x	x	x	x		
Q8DSV3	serC	Phosphoserine aminotransferase	Amino acid biosynthesis	x	x	x	x	x	x	x	x
Q8DTP2	SMU_1291c	Putative chorismate mutase	Amino acid biosynthesis						x		
Q8DUG5	SMU_965	Homoserine dehydrogenase	Amino acid biosynthesis						x		
Q8DWH9	thrC	Putative threonine synthase	Amino acid biosynthesis						x		
Q8DUL2	SMU_913	Glutamate dehydrogenase	Amino acid metabolism	x	x	x	x	x	x	x	x

Bacterial adherence and biofilm formation

Q54443	dexA	Dextranase	Adherence					x			
Q8DRV2	gbpA	Glucan-binding protein A, GbpA	Adherence	x	x	x	x	x	x	x	x
Q9KIJ3	sloC	Metal ABC transporter substrate-binding lipoprotein	Adherence	x	x	x	x	x	x	x	x
Q8DWM3	gbpB	Putative secreted antigen GbpB/SagA putative peptidoglycan hydrolase	Biofilm formation	x	x	x	x	x	x	x	x
Q8DTF1	gbpC	Glucan-binding protein C, GbpC	Biofilm formation	x							
Q8CVC7	SMU_609	Putative 40K cell wall protein	Biofilm formation	x	x	x	x	x	x	x	x
P23504	spaP	Cell surface antigen I/II	Cell wall antigen	x	x	x	x	x	x	x	x
P11701	ftf	Levansucrase	EPS biosynthesis				x				
P08987	gtfB	Glucosyltransferase-I	EPS biosynthesis				x				x

P13470	gtfC	Glucosyltransferase-SI	EPS biosynthesis	x	x	x		x	x	x	
P49331	gtfD	Glucosyltransferase-S	EPS biosynthesis	x			x	x			
Carbohydrate metabolism and energy production											
Q8DUS5	SMU_824	dTDP-4-dehydrorhamnose reductase	Carbohydrate biosynthesis	x	x	x	x	x			
Q8DTT9	rpiA	Ribose-5-phosphate isomerase A	Carbohydrate degradation		x	x	x	x		x	
Q03174	fruA	Fructan beta-fructosidase	Carbohydrate metabolic process	x	x	x	x	x	x	x	x
Q8DVV3	gapC	Glyceraldehyde-3-phosphate dehydrogenase	Carbohydrate metabolic process	x	x	x	x	x	x	x	x
Q8DT31	glgP	Alpha-1,4 glucan phosphorylase	Carbohydrate metabolic process	x	x	x	x	x	x	x	x
Q8DTC6	glmM	Phosphoglucosamine mutase	Carbohydrate metabolic process							x	
Q8DV70	nagB	Glucosamine-6-phosphate deaminase	Carbohydrate metabolic process	x		x	x				
Q59934	pfl	Formate acetyltransferase	Carbohydrate metabolic process	x	x	x	x	x	x	x	x
Q8CWY1	pfl2	Formate acetyltransferase (Pyruvate formate-lyase 2)	Carbohydrate metabolic process		x						
Q8DU72	pgm	Putative phosphoglucomutase	Carbohydrate metabolic process	x	x		x	x			
P95780	rmlB	dTDP-glucose 4,6-dehydratase	Carbohydrate metabolic process					x		x	
O68579	ppaC	Probable manganese-dependent inorganic pyrophosphatase	Energy metabolism	x	x	x	x	x	x	x	x
Q8DT52	glgB	1,4-alpha-glucan branching enzyme GlgB	Glycogen biosynthesis		x						
Q8DTS9	eno	Enolase	Glycolytic process	x	x	x	x	x	x	x	x
Q8DWG0	fbaA	Fructose-1,6-biphosphate aldolase	Glycolytic process	x	x	x	x	x	x	x	x
P59161	gpmA	2,3-bisphosphoglycerate-dependent phosphoglycerate mutase	Glycolytic process	x	x	x	x	x	x	x	x
P26283	ldh	L-lactate dehydrogenase	Glycolytic process	x	x	x	x	x	x	x	x
Q8DTX6	pfkA	ATP-dependent 6-phosphofructokinase	Glycolytic process	x	x	x	x	x	x	x	x
Q9X670	pgi	Glucose-6-phosphate isomerase	Glycolytic process	x	x	x	x	x	x		x
Q8DVV2	pgk	Phosphoglycerate kinase	Glycolytic process	x	x	x	x	x	x	x	x
Q8DTX7	pykF	Pyruvate kinase	Glycolytic process	x	x	x	x	x	x	x	x
P72484	tpiA	Triosephosphate isomerase	Glycolytic process	x	x	x	x	x	x		x
Cell division, replication and cell wall synthesis											
Q8DSX7	ftsK	DNA translocase FtsK	Cell cycle								x

Q8DVE1	ftsQ	Cell division protein DivIB	Cell cycle	x							
Q8DVL1	gpsB	Cell cycle protein GpsB	Cell cycle	x							
Q8DVQ8	SMU_412c	Putative Hit-like protein involved in cell-cycle regulation	Cell cycle			x					
Q8DVD4	divIVA	Putative cell division protein DivIVA	Cell division	x	x	x	x	x	x	x	x
Q8DVE0	ftsA	Cell division protein FtsA O	Cell division	x	x	x	x	x	x	x	x
Q8DVD9	ftsZ	Cell division protein FtsZ	Cell division	x	x	x	x		x	x	x
Q8DUE4	SMU_991	Putative ribonucleotide reductase	Cell division			x			x		
Q8DSP6	mltG	Endolytic murein transglycosylase	Cell wall biosynthesis		x						
O70055	dltC	D-alanyl carrier protein	Cell wall biosynthesis	x	x	x		x	x		x
Q8DVM5	pbp2x	Putative penicillin-binding protein 2X	Cell wall biosynthesis								
Q8DVA0	pbp2b	Penicillin-binding protein 2b	Cell wall biosynthesis			x					
Q8DT57	murA1	UDP-N-acetylglucosamine 1-carboxyvinyltransferase 1	Peptidoglycan biosynthesis	x							
Q8DST2	murE	UDP-N-acetylmuramoyl-L-alanyl-D-glutamate--L-lysine ligase	Peptidoglycan biosynthesis		x				x		
Q8DSQ5	murI	Glutamate racemase	Peptidoglycan biosynthesis			x	x		x		
Q8DV07	murM	Putative peptidoglycan branched peptide synthesis protein MurM	Peptidoglycan biosynthesis				x				
Q8DUJ1	SMU_937	Putative mevalonate diphosphate decarboxylase	Peptidoglycan biosynthesis		x						
Q8DWN8	dnaN	Beta sliding clamp	Replication			x					
Q8DT49	ligA	DNA ligase	Replication							x	
Q8DW68	SMU_207c	Putative transposon protein	Replication	x	x		x	x			
Q8DWN9	dnaA	Chromosomal replication initiator protein DnaA	Replication	x						x	
Q8DU41	gyrA	DNA gyrase subunit A	Replication	x	x	x	x	x	x	x	x
Q8DTW6	parC	DNA topoisomerase 4 subunit A	Replication							x	

Nucleoside/Nucleotide metabolism and biosynthesis

P95787	atpA	ATP synthase subunit alpha	Nucleotide biosynthesis	x	x	x	x	x	x	x	x
P95789	atpD	ATP synthase subunit beta	Nucleotide biosynthesis	x	x	x	x	x	x	x	x
Q8DRR2	guaB	Inosine-5'-monophosphate dehydrogenase	Nucleotide biosynthesis	x	x	x	x	x	x	x	x
Q8DU98	nadK	NAD kinase	Nucleotide biosynthesis	x							
Q8DWM2	prs1	Ribose-phosphate pyrophosphokinase 1	Nucleotide biosynthesis			x					

P72478	purB	adenylosuccinate lyase	Nucleotide biosynthesis	x	x	x	x	x	x		x
Q8DWJ5	purE	N5-carboxyaminoimidazole ribonucleotide mutase	Nucleotide biosynthesis	x							
Q8DUP5	pyrB	Aspartate carbamoyltransferase	Nucleotide biosynthesis				x				
Q8DTV2	pyrE	Orotate phosphoribosyltransferase	Nucleotide biosynthesis		x	x	x	x	x		
Q8DSY1	pyrH	Uridylate kinase	Nucleotide biosynthesis	x					x		
Q8DVL6	SMU_464	Nicotinate phosphoribosyltransferase	Nucleotide biosynthesis	x	x	x	x	x	x	x	x
Q8DST6	upp	Uracil phosphoribosyltransferase	Nucleotide biosynthesis				x				
Q8CVC5	SMU_1213c	Putative 5'-nucleotidase	Nucleotide catabolism	x			x				
Other metabolic processes											
Q8DWB9	adhE	Aldehyde-alcohol dehydrogenase	Alcohol metabolic process	x	x	x	x	x	x	x	x
Q8DV28	SMU_689	Lysozyme	Cell wall catabolism	x	x	x	x	x	x	x	
Q9XB21	hup	DNA-binding protein HU	Chromosome condensation	x	x	x	x	x	x	x	x
Q8DWK2	SMU_43	Putative site-specific DNA-methyltransferase restriction-modification protein	DNA methylation	x							
Q8DSN3	acp	acyl carrier protein	Fatty acid biosynthesis	x	x	x	x	x	x	x	x
Q8DSN6	fabF	3-oxoacyl-[acyl-carrier-protein] synthase 2	Fatty acid biosynthesis	x		x	x				
Q8DSN0	fabM	Trans-2-decenoyl-[acyl-carrier-protein] isomerase	Fatty acid biosynthesis		x						
Q8DSN8	fabZ	3-hydroxyacyl-[acyl-carrier-protein] dehydratase FabZ	Fatty acid biosynthesis	x				x			
Q8DTJ2	SMU_1345c	Putative peptide synthetase	Metabolic processes		x						x
Q8DS57	ackA	acetate kinase	Metabolic processes		x	x		x	x		
Q8DWD5	adhC	Dihydrolipoamide acetyltransferase component of pyruvate dehydrogenase complex	Metabolic processes		x	x	x	x	x		
Q8DTJ5	bacA1	Putative bacitracin synthetase 1 BacA	Metabolic processes	x	x		x	x			
Q8DTJ7	bacA2	Putative surfactin synthetase	Metabolic processes	x				x			
Q8DVK1	pknB	Putative serine/threonine protein kinase	Metabolic processes			x					
Q8DUA0	SMU_1043c	Putative phosphotransacetylase	Metabolic processes		x	x	x		x		x
Q8DTM7	SMU_1306c	Nucleotide-binding protein	Metabolic processes	x	x	x	x		x		x
Q8DTK0	SMU_1337c	Putative alpha/beta superfamily hydrolase	Metabolic processes					x		x	
Q8DW46	SMU_229	Uncharacterized protein	Metabolic processes			x	x		x		
Q8DWM1	SMU_24	aminotransferase	Metabolic processes	x	x	x	x	x	x	x	x

Q8DW28	SMU_251	Uncharacterized protein	Metabolic processes				x				
Q8DVE5	SMU_546	Putative GTP-binding protein	Metabolic processes	x	x	x	x	x	x	x	x
Q8DW01	tkt	Transketolase	Metabolic processes	x							
Q8DTL3	budC	Putative acetoin dehydrogenase	Oxidation-reduction process	x	x	x	x	x	x	x	x
Q8DVF0	dpr	Peroxide resistance protein Dpr	Oxidation-reduction process	x	x	x	x	x	x	x	x
Q8DTN9	flaW	Putative flavodoxin	Oxidation-reduction process	x	x	x	x	x		x	x
Q59931	gapN	NADP-dependent glyceraldehyde-3-phosphate dehydrogenase	Oxidation-reduction process	x	x	x	x	x	x	x	x
O68574	hlyX	Putative hemolysin	Oxidation-reduction process	x		x					
Q8DUA3	MU_1040c	Putative oxidoreductase, short-chain dehydrogenase/reductase	Oxidation-reduction process								x
Q8DSZ9	SMU_1602	Putative NAD(P)H-flavin oxidoreductase	Oxidation-reduction process			x					
Q8DSN4	SMU_1742c	Putative trans-2-enoyl-ACP reductase	Oxidation-reduction process		x	x	x	x	x	x	
Q8DWM7	ftsH	ATP-dependent zinc metalloprotease FtsH	Proteolysis	x	x	x	x	x	x	x	x
Q8DRQ6	htrA	Serine protease HtrA	Proteolysis			x			x		
Q8DV90	cshA	DEAD-box ATP-dependent RNA helicase CshA	RNA catabolic process	x	x	x	x	x	x	x	x
Q8DWB2	pnp	Polyribonucleotide nucleotidyltransferase	RNA degradation	x	x	x	x	x	x	x	x
Q8DV10	ppc	Phosphoenolpyruvate carboxylase	Tricarboxylic acid cycle			x			x	x	x
Transcription											
Q8DTB3	rnj	Ribonuclease J	RNA processing	x	x	x	x	x	x	x	x
Q8DT90	rnz	Ribonuclease Z	RNA processing			x					
Q8DSY7	ybeY	Endoribonuclease YbeY	RNA processing			x					
O07329	ccpA	Catabolite control protein A	Transcription	x		x	x	x	x	x	x
Q8DUH3	clp	Putative Clp-like ATP-dependent protease, ATP-binding subunit	Transcription	x	x	x	x	x	x	x	x
Q8DVD0	clpE	ATP-dependent protease ClpE	Transcription	x	x	x		x	x	x	x
I6L8Z5	covR	Putative response regulator CovR VicR-like protein	Transcription				x				
I6L8Z3	gcrR	Response regulator GcrR for glucan-binding protein C	Transcription	x	x	x	x	x	x	x	x
Q8DSP7	greA	Transcription elongation factor GreA	Transcription	x	x	x	x	x	x	x	x
Q8DVQ2	nusA	Transcription termination/antitermination protein NusA	Transcription	x	x	x	x	x	x	x	x
Q8DS82	nusG	Transcription termination/antitermination protein NusG	Transcription	x	x	x	x	x	x	x	x

P45596	ptsH	Phosphocarrier protein HPr	Transcription		x	x	x	x			x
Q8DS36	rpoA	DNA-directed RNA polymerase subunit alpha	Transcription	x	x	x	x	x	x	x	x
Q8DS46	rpoB	DNA-directed RNA polymerase subunit beta(rpoB)	Transcription	x		x	x	x	x	x	
Q8DS47	rpoC	DNA-directed RNA polymerase subunit beta'	Transcription	x	x	x	x	x	x	x	x
Q8DWG2	rpoE	Probable DNA-directed RNA polymerase subunit delta	Transcription	x	x	x	x	x	x	x	x
I6L923	sloR	Putative metal-dependent transcriptional regulator	Transcription						x		
Q8DS71	SMU_1964c	Putative response regulator	Transcription			x					
Q8DW57	SMU_218	Putative transcriptional regulator	Transcription				x				
Q8DW40	SMU_236c	Putative transcriptional regulator	Transcription			x					
Q8DVK3	sunL	Putative RNA-binding Sun protein possible rRNA methylase	Transcription						x		
Translation and protein synthesis											
Q8CWY0	alaS	Alanine--tRNA ligase	Protein biosynthesis	x	x		x	x		x	
Q8DTM2	asnS	Asparagine--tRNA ligase	Protein biosynthesis					x	x		
Q8DRV9	aspS2	Aspartate--tRNA ligase 2	Protein biosynthesis	x			x				
Q8DVX9	gltX	Glutamate--tRNA ligase	Protein biosynthesis				x				
Q8DVD3	ileS	Isoleucine--tRNA ligase	Protein biosynthesis	x	x	x	x	x	x		x
Q8CWX2	pheT	Phenylalanine--tRNA ligase beta subunit	Protein biosynthesis	x							
Q8DSJ9	proS	Proline--tRNA ligase	Protein biosynthesis		x	x	x	x		x	
Q8DS21	rpmC	50S ribosomal protein L29	Protein biosynthesis	x		x	x	x	x		x
Q8DS44	tyrS	Tyrosine--tRNA ligase	Protein biosynthesis						x		
Q8DWN6	ychF	Ribosome-binding ATPase YchF	Protein biosynthesis	x							
Q8DUI0	clpX	ATP-dependent Clp protease ATP-binding subunit ClpX	Protein folding	x	x	x	x	x	x	x	
O06942	dnaK	Chaperone protein DnaK	Protein folding	x	x	x	x	x	x	x	x
Q8CWW6	groL	60 kDa chaperonin	Protein folding	x	x	x	x	x	x	x	x
Q8CWW5	groS	10 kDa chaperonin	Protein folding	x	x	x	x	x	x	x	x
O06941	grpE	Protein GrpE	Protein folding	x	x	x	x	x	x		x
Q8CVC6	prsA	Foldase protein PrsA	Protein folding			x					
Q8DVJ7	SMU_488	Putative hydrolase	Protein folding				x				

Q8CWZ6	tig	Trigger factor	Protein folding	x	x	x	x	x	x	x	x
Q54431	ffh	Signal recognition particle protein	Protein targeting	x				x	x		
Q8DTM5	aldR	Putative translation initiation inhibitor aldR regulator-like protein	Translation		x						
Q8DWC2	def	Peptide deformylase	Translation	x	x	x	x	x	x	x	x
Q8DSE7	efp	Elongation factor P	Translation	x							
Q8DSY2	frr	Ribosome-recycling factor	Translation	x	x	x	x	x	x		x
Q8DVV4	fusA	Elongation factor G	Translation	x	x	x	x	x	x	x	x
Q8DSG5	gatA	Glutamyl-tRNA(Gln) amidotransferase subunit A	Translation		x						
Q8DSG6	gatB	Aspartyl/glutamyl-tRNA(Asn/Gln) amidotransferase subunit B	Translation				x		x		
Q8DSG4	gatC	Aspartyl/glutamyl-tRNA(Asn/Gln) amidotransferase subunit C	Translation	x						x	
Q8DVI8	hpf	Ribosome hibernation promoting factor	Translation	x	x	x	x	x	x	x	x
Q8DS34	inf	Translation initiation factor IF-1	Translation				x				
Q8DVP9	infB	Translation initiation factor IF-2	Translation	x	x	x	x	x	x	x	x
Q8DV22	infC	Translation initiation factor IF-3	Translation				x			x	
Q8DU64	prfA	Peptide chain release factor 1	Translation				x				
Q8DSY0	rplA	50S ribosomal protein L1	Translation	x	x	x	x	x	x	x	x
G1XVB9	rplB	50S ribosomal protein L2	Translation	x	x	x	x	x	x	x	x
Q8DS16	rplC	50S ribosomal protein L3	Translation	x	x	x	x	x	x		x
Q8DS17	rplD	50S ribosomal protein L4	Translation	x	x	x	x	x	x	x	x
Q8DS25	rplE	50S ribosomal protein L5	Translation	x	x	x	x	x	x	x	x
Q8DS28	rplF	50S ribosomal protein L6	Translation	x	x	x	x	x	x	x	x
Q8DRS8	rplI	50S ribosomal protein L9	Translation	x	x	x	x	x	x	x	x
Q8DUH2	rplJ	50S ribosomal protein L10	Translation	x	x	x	x	x	x	x	x
Q8DSX9	rplK	50S ribosomal protein L11	Translation	x	x	x	x	x	x	x	x
Q8DUG9	rplL	50S ribosomal protein L7/L12	Translation	x	x	x	x	x	x	x	x
Q8DW98	rplM	50S ribosomal protein L13	Translation	x	x	x	x	x	x	x	x
Q8DS23	rplN	50S ribosomal protein L14	Translation	x	x	x	x	x	x	x	x
Q8DS31	rplO	50S ribosomal protein L15	Translation	x	x	x	x	x	x	x	x
Q8DS37	rplQ	50S ribosomal protein L17	Translation	x	x	x	x	x	x	x	x

Q8DS29	rplR	50S ribosomal protein L18	Translation	x	x	x	x	x	x	x	x
Q8DTP5	rplS	50S ribosomal protein L19	Translation					x			
Q8DUQ6	rplU	50S ribosomal protein L21	Translation	x	x	x	x	x	x	x	x
Q8DS19	rplV	50S ribosomal protein L22	Translation	x	x	x		x	x	x	x
G1XVC0	rplW	50S ribosomal protein L23	Translation	x	x		x	x	x	x	x
Q8DS24	rplX	50S ribosomal protein L24	Translation	x	x	x	x	x	x	x	x
Q8DUQ4	rpmA	50S ribosomal protein L27	Translation	x	x	x	x	x	x	x	x
Q8DWE1	rpmB	50S ribosomal protein L28	Translation	x							
Q8DS30	rpmD	50S ribosomal protein L30	Translation			x	x	x	x		
Q8DTN5	rpmE2	50S ribosomal protein L31 type B	Translation	x	x	x	x	x	x	x	x
Q8DS11	rpsB	30S ribosomal protein S2	Translation	x	x	x	x	x	x	x	x
P59186	rpsC	30S ribosomal protein S3	Translation	x	x	x	x	x	x	x	x
P59133	rpsD	30S ribosomal protein S4	Translation	x	x	x	x	x	x	x	x
P59125	rpsE	30S ribosomal protein S5	Translation	x	x	x	x	x	x	x	x
Q8DSD7	rpsF	30S ribosomal protein S6	Translation	x	x	x	x	x	x	x	x
Q8DVV5	rpsG	30S ribosomal protein S7	Translation	x	x			x	x		
Q8DS27	rpsH	30S ribosomal protein S8(	Translation	x	x	x	x	x	x	x	x
Q8DW97	rpsI	30S ribosomal protein S9	Translation					x			
P48853	rpsJ	30S ribosomal protein S10	Translation	x	x	x	x	x	x	x	x
P59378	rpsK	30S ribosomal protein S11	Translation	x	x	x	x	x	x	x	x
Q8DS35	rpsM	30S ribosomal protein S13	Translation	x	x	x	x	x	x	x	x
Q8DWB3	rpsO	30S ribosomal protein S15	Translation	x	x	x	x	x	x	x	x
Q8DUN9	rpsP	30S ribosomal protein S16	Translation	x				x			
Q8DS22	rpsQ	30S ribosomal protein S17	Translation	x	x	x					
Q8DS18	rpsS	30S ribosomal protein S19	Translation	x	x	x	x	x	x	x	
Q8DTW9	rs1	Putative ribosomal protein S1 sequence specific DNA-binding protein	Translation	x	x	x	x	x	x	x	x
Q8DS12	tsf	Elongation factor Ts	Translation	x	x	x	x	x	x	x	x
P72483	tuf	Elongation factor Tu	Translation	x	x	x	x	x	x	x	x

Transport											
Q8DT63	glnQ	Putative amino acid ABC transporter, ATP-binding protein	Amino acid transport	x	x		x	x	x	x	
Q8DU84	opuAa	Putative ABC transporter, ATP-binding protein, proline/glycine betaine transport system	Amino acid transport					x			
I6L912	SMU_1942c	Putative amino acid binding protein	Amino acid transport			x					
Q8DUT1	SMU_815	Putative amino acid transporter, amino acid-binding protein	Amino acid transport						x		
Q8DUS9	SMU_817	Putative amino acid transporter, amino acid-binding protein	Amino acid transport	x							
Q8DSZ8	lguL	Putative lactoylglutathione lyase	Metal ion binding	x	x	x	x	x	x	x	x
Q8DSF0	secA	Protein translocase subunit SecA	Protein transport				x			x	
Q8DUN3	fruI	Inducible fructose permease	Sugar transport		x				x	x	x
Q00752	msmK	Multiple sugar-binding transport ATP-binding protein MsmK	Sugar transport	x			x				
Q8DT01	ptcB	Putative PTS system, cellobiose-specific IIB component	Sugar transport			x					
Q8DSC4	ptnA	Putative PTS system, mannose-specific component IIAB	Sugar transport		x		x	x		x	x
Q8DS05	ptsG	Putative PTS system, glucose-specific IIABC component	Sugar transport	x	x		x	x	x	x	x
P45595	ptsI	Phosphoenolpyruvate-protein phosphotransferase	Sugar transport	x	x	x	x	x	x	x	x
I6L910	ptxB	Putative PTS system, enzyme IIB component	Sugar transport				x				
P12655	scrA	PTS system sucrose-specific EIIBCA component	Sugar transport	x					x		
Q8DWF9	SMU_100	Putative sorbose PTS system, IIB component	Sugar transport						x		
Q8DS75	SMU_1958c	Putative PTS system, mannose-specific IIC component	Sugar transport	x	x	x		x	x	x	
Q8DS74	SMU_1960c	Putative PTS system, mannose-specific IIB component	Sugar transport	x	x	x	x	x	x	x	x
Q8DS73	SMU_1961c	Putative PTS system, sugar-specific enzyme IIA component	Sugar transport	x	x	x	x	x	x	x	x
P95786	atpH	ATP synthase subunit delta	Transport	x			x	x	x	x	x
I6L926	lemA	Putative cytoplasmic membrane protein LemA-like protein	Transport		x						
Q8DSU0	livK	Putative ABC transporter, branched chain amino acid-binding protein	Transport	x	x	x	x	x	x	x	x
Q8DUY5	lmrB	Putative drug-export protein multidrug resistance protein	Transport		x						
Q8DW25	oppA	Putative oligopeptide ABC transporter, substrate-binding protein OppA	Transport	x	x	x	x	x	x	x	x
Q8DU85	opuAb	Putative ABC transporter, proline/glycine betaine permease protein	Transport								x
I6L8Z4	rgpC	Transport permease protein	Transport		x						
Q8DW86	sloA	Putative ABC transporter, ATP-binding protein possible iron and/or manganese ABC transport system	Transport			x				x	x
Q8DUA2	SMU_1041	Putative ABC transporter, ATP-binding protein	Transport					x		x	x

Q8DU57	SMU_1093	Putative ABC transporter, permease protein	Transport	x			x	x			
Q8DU36	SMU_1121c	Putative ABC transporter	Transport	x	x	x	x	x	x		x
Q8DTZ4	SMU_1166c	Putative ABC transporter, permease protein	Transport	x							
Q8CM14	SMU_1348c	Putative ABC transporter ATP-binding protein	Transport				x				
Q8DTD8	SMU_1412c	Putative ABC transporter, membrane protein subunit and ATP-binding protein	Transport	x							
Q8DT62	SMU_1520	Putative ABC transporter, glutamine binding protein	Transport	x	x	x	x	x	x	x	x
Q8DW32	SMU_247	Putative ABC transporter ATP-binding protein	Transport	x	x	x	x	x	x	x	x
Q8DW31	SMU_248	Putative ABC transporter, membrane protein	Transport				x	x		x	
Q8DW05	SMU_286	Putative ABC transporter ATP-binding protein ComA	Transport					x			
Q8DUT7	SMU_806c	Putative glutamine ABC transporter, permease protein	Transport	x	x		x	x	x		
Q8DUD7	SMU_998	Putative ABC transporter, periplasmic ferrichrome-binding protein	Transport	x	x	x	x	x	x	x	x
Q8DT34	trkB	Putative potassium uptake system protein TrkB	Transport					x			
Uncharacterized/Unknown											
Q8DVH8	MU_510c	Uncharacterized protein	Uncharacterized				x				
Q8DU92	SMU_1052	Uncharacterized protein	Uncharacterized	x							
Q8DU59	SMU_1090	Uncharacterized protein	Uncharacterized			x					
Q8DU49	SMU_1104c	Uncharacterized protein	Uncharacterized			x					
Q8DU43	SMU_1111c	Uncharacterized protein	Uncharacterized						x		
Q8DTW8	SMU_1201c	Uncharacterized protein	Uncharacterized	x	x	x	x	x	x	x	
Q8DTG7	SMU_1377c	Uncharacterized protein	Uncharacterized	x	x	x	x		x		x
Q8DTB5	SMU_1442c	Uncharacterized protein	Uncharacterized	x	x	x	x	x	x	x	x
Q8DT86	SMU_1479	Uncharacterized protein	Uncharacterized	x		x	x	x	x	x	x
Q8DSW7	SMU_1641c	Uncharacterized protein	Uncharacterized	x	x	x	x	x	x	x	x
Q8DSS8	SMU_1681c	Uncharacterized protein	Uncharacterized					x			
Q8DSM0	SMU_1760c	Uncharacterized protein	Uncharacterized	x	x	x	x	x	x	x	x
Q8DSI7	SMU_1798c	Uncharacterized protein	Uncharacterized						x		
Q8DSE8	SMU_1846c	Uncharacterized protein	Uncharacterized	x	x	x	x	x	x		x
Q8DSA4	SMU_1904c	Uncharacterized protein	Uncharacterized	x		x	x	x	x		x

I6L8Z9	SMU_1925c	Uncharacterized protein	Uncharacterized	x						x	
Q8DW70	SMU_205c	Uncharacterized protein	Uncharacterized				x				
Q8DRX7	SMU_2079c	UPF0297 protein	Uncharacterized	x	x	x	x	x	x	x	x
Q8DW66	SMU_209c	Uncharacterized protein	Uncharacterized	x	x	x	x	x	x	x	x
Q8DW65	SMU_210c	Uncharacterized protein	Uncharacterized	x	x	x	x	x	x		x
Q8DRQ9	SMU_2160	Uncharacterized protein	Uncharacterized			x			x		
Q8DW41	SMU_235	Uncharacterized protein	Uncharacterized	x	x	x	x	x	x	x	x
Q8DVS4	SMU_393	Uncharacterized protein	Uncharacterized	x		x	x	x	x	x	x
Q8DVC8	SMU_564	Uncharacterized protein	Uncharacterized	x	x	x	x	x	x	x	x
Q8DWI5	SMU_63c	Uncharacterized protein	Uncharacterized						x	x	
Q8DV31	SMU_685	Uncharacterized protein	Uncharacterized	x	x	x	x		x		
Q8DUY9	SMU_739c	Uncharacterized protein	Uncharacterized		x				x		x
Q8DUY0	SMU_751	Uncharacterized protein	Uncharacterized	x				x	x	x	
Q8DUX2	SMU_768	Uncharacterized protein	Uncharacterized			x		x		x	
Q8DUP2	SMU_862	Uncharacterized protein	Uncharacterized							x	
Q8DTJ0	SMU_1347c	Uncharacterized protein	Unknown			x		x			x
Q8DRT3	SMU_2133c	Putative membrane protein	Unknown			x					
Q8DVU6	SMU_369c	UPF0356 protein	Unknown							x	
Q8DVN2	SMU_447	UPF0291 protein	Unknown	x			x				x
Q8DWH8	SMU_72	UPF0237 protein	Unknown	x	x	x	x	x	x	x	x
Q8DWH7	SMU_73	UPF0210 protein	Unknown							x	
Q8DUV9	SMU_782	UPF0342 protein	Unknown	x	x	x	x	x	x	x	x
Q8DUV0	SMU_793	Uncharacterized protein	Unknown		x			x	x		x
